# TREM2 Deficiency Regulates Macrophage Apoptosis and Repair in Radiation-Induced Skin Injury

**DOI:** 10.34133/research.1018

**Published:** 2025-12-04

**Authors:** Zijian Chen, Siyuan Cai, Pengfei Li, Ziyi Zhou, Zhenxing Huang, Juntao Deng, Linbo Jin, Zucheng Luo, Dongli Fan, Junli Zhou, Fazhi Qi, Yiming Zhang

**Affiliations:** ^1^ Department of Plastic and Cosmetic Surgery, Xin Qiao Hospital, Army Medical University, Chongqing 400037, China.; ^2^ Department of Plastic and Reconstructive Surgery, Zhongshan Hospital, Fudan University, Shanghai, China.; ^3^ Department of Gastrointestinal Surgery, Shanghai 4th People’s Hospital, Tongji University, Shanghai 200081, China.; ^4^ Department of Burn and Plastic Surgery, The Tenth Affiliated Hospital, Southern Medical University (Dongguan People’s Hospital), Dongguan, China.

## Abstract

Radiation-induced skin injury (RISI) is a common and debilitating complication of radiotherapy, characterized by persistent inflammation and delayed wound healing. Macrophages play a central role in this process; however, the molecular mechanisms governing their dysfunction under radiation stress remain poorly understood. To elucidate the role of triggering receptor expressed on myeloid cells 2 (TREM2) in macrophage regulation after irradiation, we combined single-cell RNA sequencing, in vivo mouse models, and in vitro macrophage assays. Conditional knockout mice (*LysM*^Cre^*Trem2*^flox/flox^) were used to selectively delete *Trem2* in macrophages. Radiation induced a distinct TREM2^+^ macrophage subset; however, despite elevated *Trem2* mRNA, protein levels declined due to ADAM17-mediated shedding driven by radiation-induced reactive oxygen species (ROS) accumulation and NRF2 activation. Inhibition or small interfering RNA (siRNA)-mediated knockdown of ADAM17 restored TREM2 protein expression, reduced soluble TREM2 release, improved macrophage survival, and promoted anti-inflammatory M2 polarization. Conversely, *Trem2* deficiency enhanced apoptosis, sustained inflammation, and delayed wound healing, whereas *Trem2* overexpression or local adoptive transfer of TREM2^+^ macrophages accelerated tissue repair. Mechanistically, TREM2 conferred radioprotection through extracellular signal-regulated kinase (ERK) pathway activation, linking the ROS–NRF2–ADAM17 axis to TREM2–ERK signaling in macrophage survival and polarization. Collectively, these findings identify a novel regulatory cascade, ROS–NRF2–ADAM17–TREM2–ERK, that governs macrophage fate under irradiation. Targeting this pathway or supplementing TREM2^+^ macrophages may offer promising therapeutic strategies for mitigating RISI.

## Introduction

The use of ionizing radiation has both beneficial and harmful effects [1]. It is a fundamental modality in oncological treatment, offering curative potential for specific neoplasms and providing long-term clinical benefits [2]. However, improper handling or accidental leakage of nuclear materials can lead to catastrophic outcomes, including acute radiation syndrome (ARS) and severe organ dysfunction. When radiation-induced injury is confined to the skin or the radiation dose is insufficient to penetrate deeper tissues, the condition is termed radiation-induced skin injury (RISI) [3–5]. RISI not only can result from nuclear leaks or weaponry but also is a common side effect of radiotherapy. In the context of tumor radiotherapy, the incidence of RISI is nearly ubiquitous, with 85% to 95% of patients experiencing varying degrees of skin damage [6], which can substantially impair quality of life and treatment outcomes [4].

The molecular mechanisms underlying RISI are complex, involving both direct DNA damage and subsequent cellular responses [7,8]. The immune response is a critical component of RISI progression, as immune cells undergo marked changes during this process [9]. Acute radiation-induced skin toxicity is associated with elevated levels of various cytokines and chemokines, particularly interleukin-1α (IL-1α), IL-1β, tumor necrosis factor-α (TNF-α), IL-6, IL-8, CCL4, CXCL10, and CCL2 [9–12]. Macrophages play a central role in these inflammatory responses. Traditionally, macrophages are categorized into 2 polarization states: the classically activated M1 phenotype, which is proinflammatory, and the alternatively activated M2 phenotype, which promotes inflammation resolution and tissue repair [13]. Recent advances in single-cell analysis have provided a more nuanced understanding of macrophage heterogeneity and polarization [14], showing that macrophage activation spans a spectrum of states with distinct functional roles across diseases.

Among these subsets, macrophages expressing *Trem2* (triggering receptor expressed on myeloid cells 2; protein: TREM2) have emerged as a unique population. Initially studied in neurodegenerative diseases such as Alzheimer’s, TREM2 has been shown to regulate lipid metabolism, phagocytosis, and microglial survival [15–19]. Subsequent studies extended its relevance to fibrotic diseases, including liver and lung fibrosis, where TREM2^+^ macrophages act as transitional populations mediating tissue remodeling [17]. Moreover, TREM2 has been implicated in cancer and metabolic disorders, where it influences tumor immune evasion and adipose tissue homeostasis [20–22]. More recently, TREM2 has been found to contribute to cutaneous wound healing [23]. For example, TREM2 promotes skin angiogenesis through glycolytic reprogramming of macrophages via the Akt/mTOR (mammalian target of rapamycin)/hypoxia-inducible factor-1α (HIF-1α) axis [24], whereas loss of TREM2 exacerbates inflammation and delays repair in diabetic wounds [25].

Despite these advances, it remains unclear how TREM2^+^ macrophages respond to radiation-induced damage and whether they contribute to the unique pathology of radiation-impaired wound healing. Elucidating their behavior and function under radiation injury not only extends existing wound-healing research but also addresses an important clinical gap.

This study aimed to investigate the involvement of TREM2-expressing macrophages in RISI and their role in wound healing, with a specific focus on the kinetics of TREM2 expression during radiation-impaired wound repair.

## Results

### Integration of single-cell RNA sequencing and bulk RNA sequencing identifies radiation-associated *Trem2* expression

**Fig. 1.**
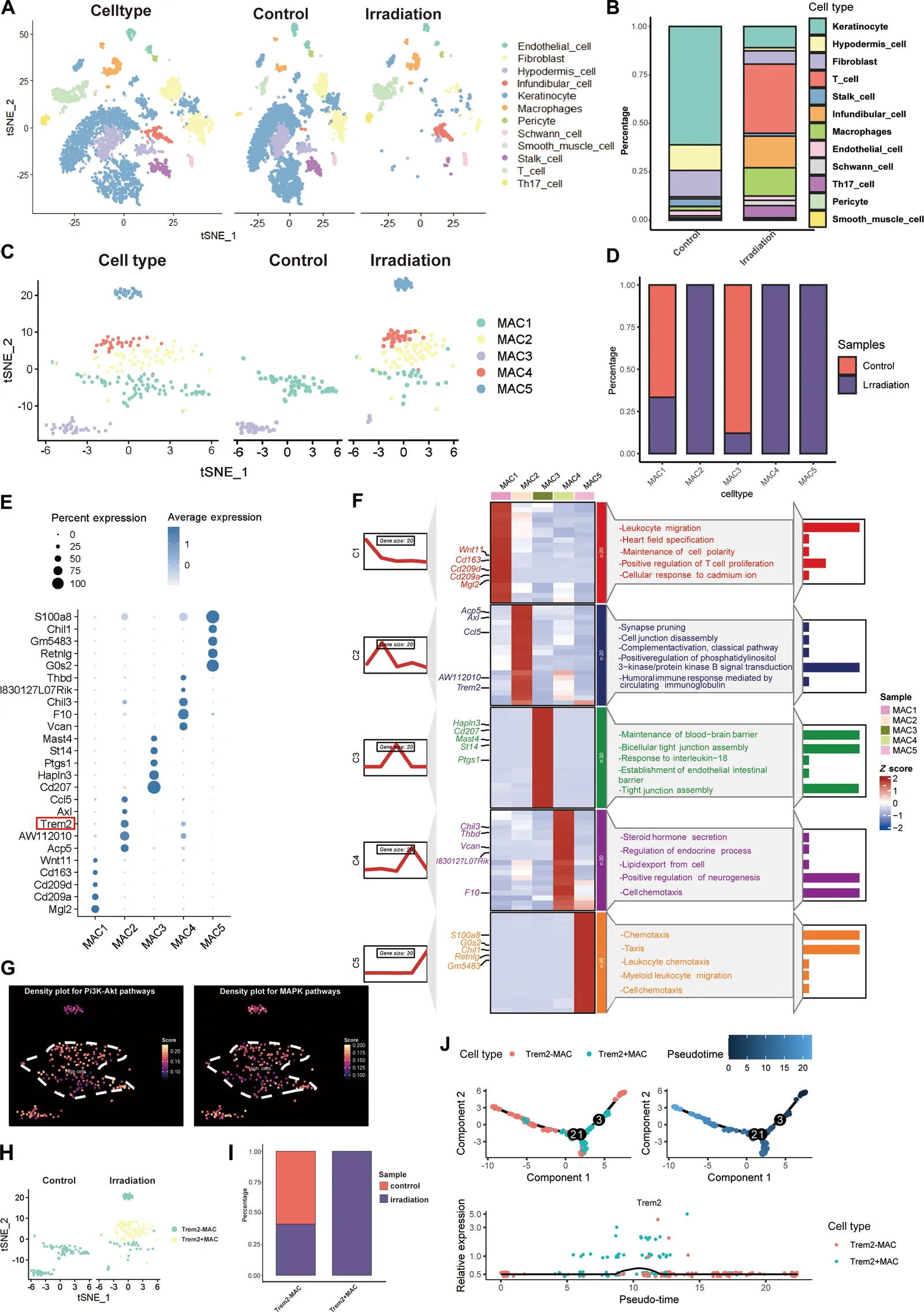
scRNA-seq reveals irradiation-induced *Trem2* expression in skin macrophages. (A) tSNE plots showing cell type annotation based on CellMarker 2.0, and distribution of cell clusters in control and irradiated groups. (B) Proportion of each cell population comparing control and irradiation groups. (C) Annotation of macrophage-related clusters using marker genes. (D) Quantification of macrophage cluster ratios between control and irradiated groups. (E) Dotplot showing the expression of representative genes in macrophage subclusters, highlighting *Trem2*-specific enrichment. (F) irGSEA of macrophage clusters, including a gene expression heatmap and GSEA plot. (G) AUCell analysis indicating high activity of the PI3K-Akt pathway in MAC2 macrophage subclusters. (H) Separation of macrophage clusters based on *Trem2* expression. (I) Comparison of the ratio of *Trem2*-positive and *Trem2*-negative clusters between control and irradiated samples. (J) Pseudotime trajectory analysis illustrating the developmental progression of *Trem2*-positive and Trem2-negative macrophages.

To explore the macrophage subsets involved in RISI, we analyzed single-cell RNA sequencing (scRNA-seq) data derived from whole skin tissues obtained on day 14 from mice that had received 15-Gy total body irradiation and from blank controls. As shown in Fig. 1A, all cells clustered into 12 distinct populations based on canonical markers (Fig. S1A). Irradiation significantly altered cellular proportions, with increased abundance of immune cells (e.g., T cells, macrophages, and Th17 cells) (Fig. 1B).

Cell differential interaction strength analysis indicated that radiation markedly enhanced communications between immune cells, particularly macrophages, and stromal cells via inflammatory (MIF, SPP1, and CCL) [26–28] and chemotactic (CXCL) pathways [29] while weakening interactions among stromal cells in growth factor-related pathways [fibroblast growth factor (FGF), platelet-derived growth factor (PDGF), and bone morphogenetic protein (BMP)] [30,31]. Network topology analysis further identified macrophages as central hubs of these strengthened interactions, underscoring their pivotal role in the cellular response to radiation (Fig. S1B and C).

In parallel, Gene Set Enrichment Analysis (GSEA) and UCell analysis revealed the up-regulation of inflammatory pathways in macrophage clusters (Fig. S1D and E), consistent with earlier findings and supporting radiation-activated immune responses in the skin [32]. We subsequently isolated macrophage clusters for subpopulation analysis, identifying 5 subsets (MAC1 to MAC5; Fig. 1C). Comparative analysis between blank control and irradiated groups showed that MAC2, MAC4, and MAC5 were radiation-specific (Fig. 1D and E). Differential gene expression analysis highlighted elevated *Trem2* expression in MAC2 cells, and Gene Ontology (GO) enrichment/UCell analysis indicated significant phosphatidylinositol 3-kinase (PI3K) and mitogen-activated protein kinase (MAPK) pathway activation in this subset (Fig. 1F and G), implicating it in inflammation and macrophage polarization [33].

Further stratification of macrophages into *Trem2*^+^ and *Trem2*^−^ subsets confirmed radiation-induced expansion of *Trem2*^+^ macrophages. Pseudotime analysis delineated dynamic *Trem2* expression during cellular differentiation (Fig. 1H to J). Integration of *Trem2*^+^ and *Trem2*^−^ macrophages into whole-cell clusters (Fig. S2A), followed by CellChat analysis, revealed that *Trem2*^+^ macrophages displayed more frequent and stronger intercellular interactions, particularly enriched in specific signaling pathways, compared with *Trem2*^−^ subsets (Fig. S2B to E).

To extend these observations, bulk RNA-seq was analyzed in a murine excisional wound model exposed to irradiation. *Trem2* expression progressively increased at days 3, 6, and 9 compared with day 0 and controls (Fig. S3A). GSEA showed up-regulation of inflammatory response pathways at days 3 and 6 (*P* < 0.05, adjusted *P* < 0.05), with a declining but nonsignificant trend at day 9 (*P* > 0.05, adjusted *P* > 0.05). Positive regulation of reactive oxygen species (ROS) metabolic processes was enriched at all time points, reaching significance at day 6 (*P* < 0.05, adjusted *P* < 0.05), while enrichment at days 3 and 9 did not reach adjusted significance despite nominal *P* < 0.05 (Fig. S3B to D).

Differentially expressed genes (DEGs) identified at each time point (Figs. 4A, 5A, and 6A) highlighted macrophage-related functions. GO terms indicated activation of migration, proliferation, differentiation, and apoptosis programs, with *Trem2* consistently up-regulated (Figs. S4B, S5B, and S6B). GSEA further demonstrated enrichment of inflammatory responses across all time points, and of ROS metabolic processes and apoptosis at days 3 and 6 (*P* < 0.05, adjusted *P* < 0.05) (Figs. S4C to E, S5C to E, and S6C to E). Myeloid cell functional activation was also significantly enhanced (Figs. S4F, S5F, and S6F).

Finally, integration of DEGs across all groups identified 1,116 hub genes, including *Trem2*, which was markedly up-regulated (Fig. S7A). GSEA of these hub genes emphasized inflammatory pathways, notably IL-6–Janus kinase (JAK)–signal transducer and activator of transcription 3 (STAT3) and TNF-α signaling (Fig. S7B). The top 5 *Trem2*-associated GSEA signatures converged on inflammatory response and myeloid functions (Fig. S7C).

Taken together, single-cell and bulk RNA-seq analyses consistently highlight *Trem2* as a central regulator of macrophage activation and inflammatory signaling in RISI.

### Radiation impairs wound healing and modulates Trem2 expression

**Fig. 2.**
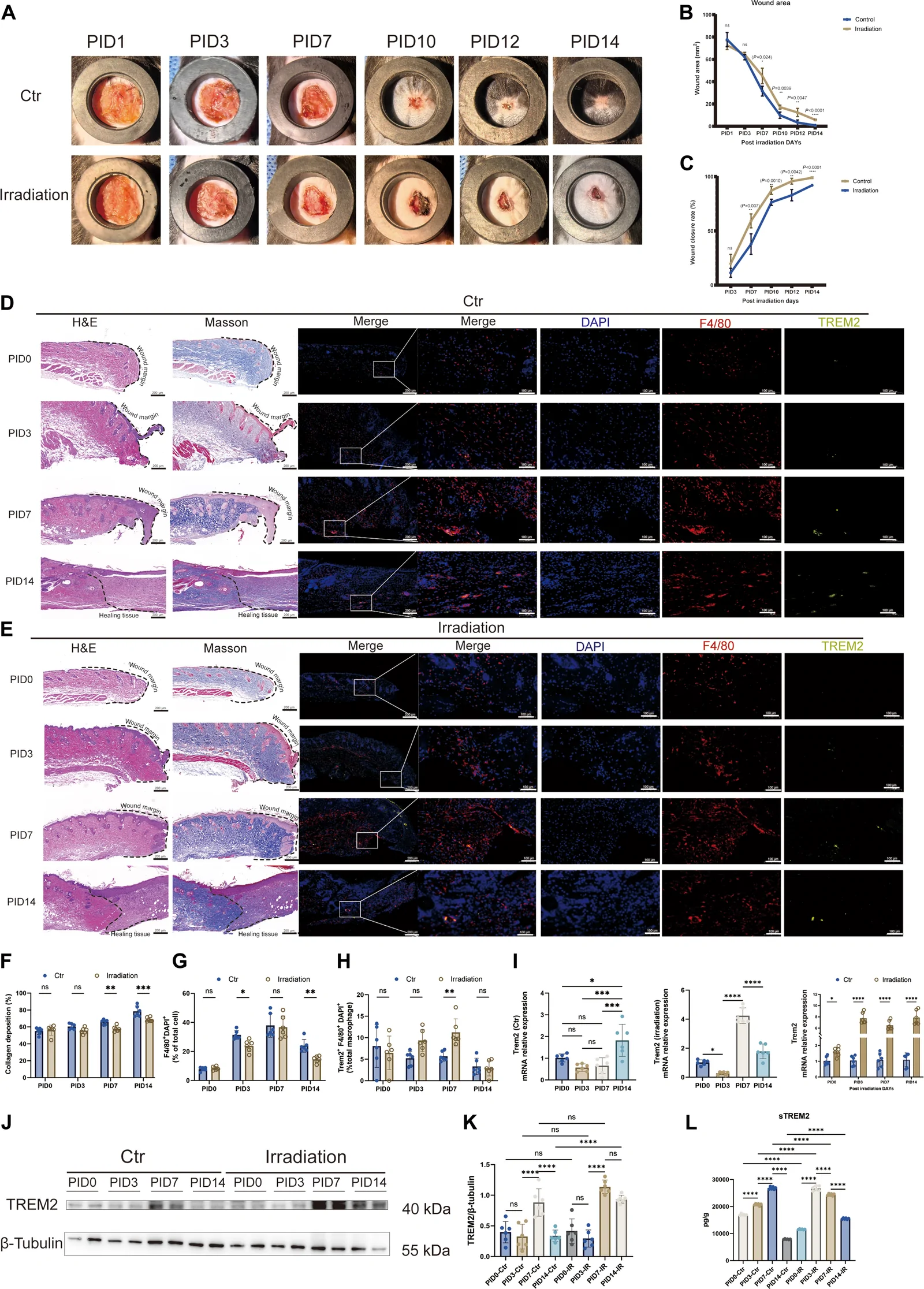
Radiation delayed wound healing in radiation-induced skin injury (RISI) and induced TREM2 expression. (A) The RISI mouse model: WT mice received 5-Gy total body x-ray irradiation followed by full-thickness excisional skin wounding (black circle, 12-mm inner diameter). (B) Representative wound area measurements (*n* = 6). (C) Quantification of wound closure rates at various time points relative to post-injury day 0 (PID0) (*n* = 6). (D and E) H&E and Masson staining of wound tissue sections at PID0, 3, 7, and 14, showing regions close to the wound margin; IF costaining of TREM2 (gold), F4/80 (red), and 4′,6-diamidino-2-phenylindole (DAPI) (blue) to identify TREM2^+^ macrophages in control and irradiated groups (scale bar was shown on panel). (F) Quantification of collagen deposition based on Masson staining (*n* = 6). (G) Total macrophage ratio calculated as the proportion of F4/80^+^DAPI^+^ cells among total DAPI^+^ cells by immunohistochemistry (*n* = 6). (H) TREM2^+^ macrophage ratio calculated as the proportion of TREM2^+^F4/80^+^DAPI^+^ cells among total F4/80^+^DAPI^+^ macrophages (*n* = 6). (I) qPCR analysis of Trem2 mRNA expression at different time points (*n* = 6). For within-group comparisons, expression was normalized to day 0 of each group. For between-group comparisons, expression levels in the irradiation group were normalized to the corresponding control group at each time point (*n* = 6). (J and K) Western blotting and analysis of TREM2 protein expression across time points in control and irradiated groups (*n* = 6). (L) ELISA measurement of soluble TREM2 (sTREM2) levels in skin tissue at different time points (*n* = 6). (*P < 0.05, **P < 0.01, ***P < 0.001, ****P < 0.0001; ns, not significant.)

Based on the multi-omics analyses that identified *Trem2*^+^ macrophages as a radiation-associated subset with inflammatory and signaling activation, we next sought to validate these findings in vivo. To this end, we established a RISI model by creating full-thickness excision wounds on the dorsal skin of wild-type (WT) mice followed by 5-Gy total body irradiation. Initial observations revealed that although radiation exposure did not significantly impair wound healing during the first 3 d, a marked deceleration in wound closure was evident from days 7 to 14 compared with the blank control group, as demonstrated by wound area measurements and closure rate analyses (Fig. 2A to C).

Subsequent analysis using hematoxylin and eosin (H&E) and Masson’s trichrome staining showed persistent inflammatory cell accumulation at wound margins, accompanied by reduced collagen deposition and disorganized fiber architecture on days 7 and 14 post-injury (Fig. 2D to F). Consistent with scRNA-seq and bulk RNA-seq findings, immunofluorescence (IF) costaining demonstrated dynamic macrophage changes, validated through F4/80 and TREM2 costaining (Fig. 2D and E). The overall macrophage ratio (F4/80^+^DAPI^+^/total DAPI^+^) decreased on days 3 and 14, whereas the TREM2^+^ macrophage subset (TREM2^+^F4/80^+^DAPI^+^/F4/80^+^DAPI^+^) showed a specific increase on day 7 (Fig. 2G and H).

Interestingly, quantitative polymerase chain reaction (qPCR) analysis confirmed time-dependent *Trem2* mRNA up-regulation on day 3 and sustained elevation followed by down-regulation on day 14 in irradiated wounds. In contrast, protein analysis via Western blotting revealed no corresponding increase in TREM2 protein on day 3. This discrepancy was resolved when enzyme-linked immunosorbent assay (ELISA) of tissue suspension uncovered early elevation of soluble TREM2 (sTREM2) on day 3 (*P* < 0.05), suggesting that radiation may influence TREM2 expression during the initial wound-healing phase (Fig. 2I to L). Taken together, these in vivo findings corroborate the multi-omics results, supporting the view that TREM2^+^ macrophages are dynamically regulated in response to radiation and may contribute to impaired wound repair.

### Effects of radiation on RAW264.7 macrophages

**Fig. 3.**
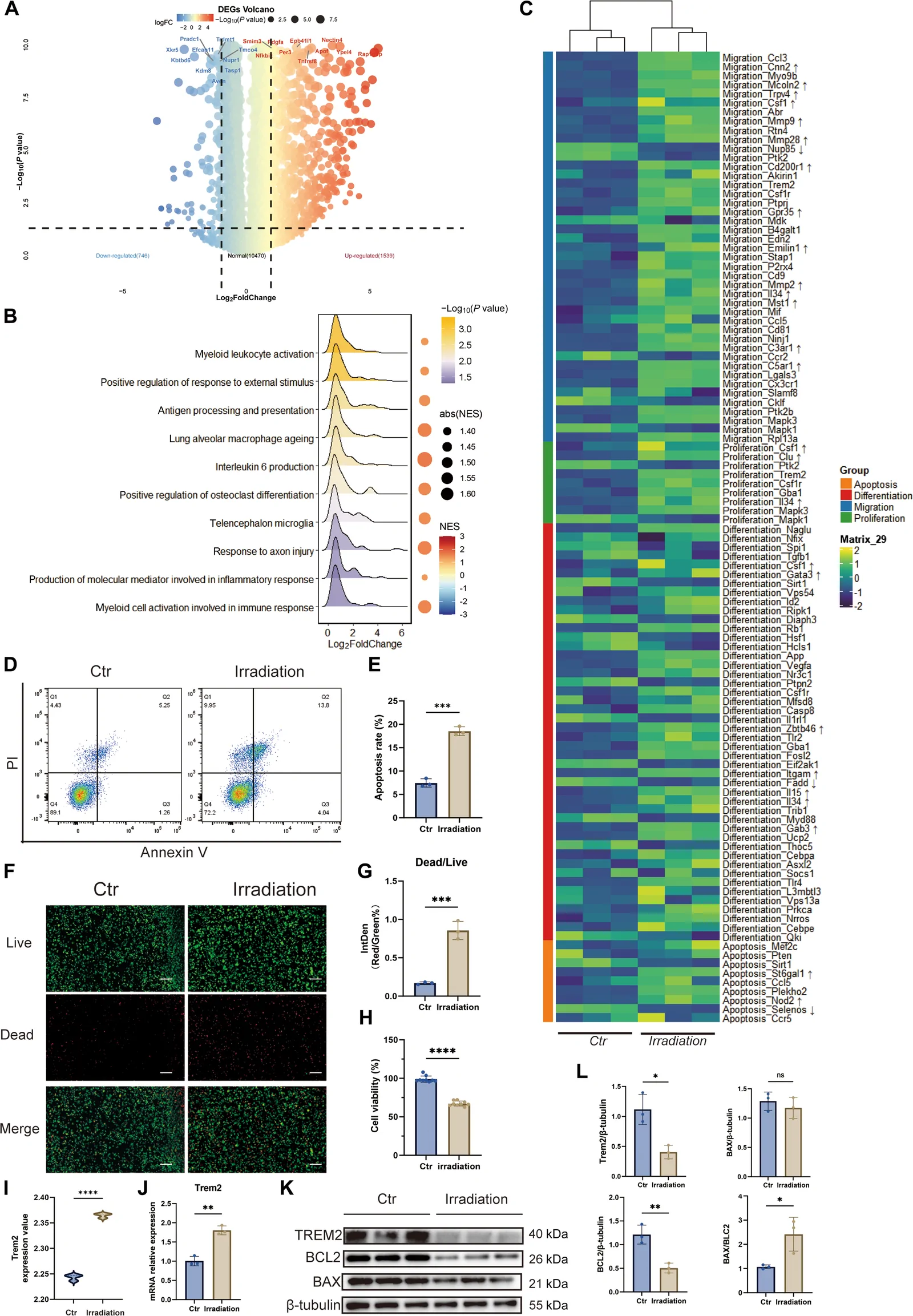
Radiation reduced RAW264.7 cell viability and induced Trem2 expression. (A) Volcano plot showing DEGs between irradiation and control RAW264.7 cells based on RNA-seq (*n* = 3). (B) GSEA identifying *Trem2*-associated pathways enriched or suppressed following irradiation. (C) Heatmap of representative genes involved in macrophage differentiation, proliferation, migration, and apoptosis from GSEA-identified pathways. Significantly up-regulated and down-regulated genes are marked with upward (↑) and downward (↓) arrows, respectively. (D and E) Flow cytometry analysis of apoptosis using Annexin V and propidium iodide (PI) staining. Early and late apoptotic cells were quantified from Q2 and Q3 quadrants (*n* = 3). (F and G) Live (green)/dead (red) staining; dead cell proportion calculated based on integrated fluorescence density (*n* = 3; scale bar, 200 μm). (H) CCK-8 assay shown relative cell viability at 24 h post-irradiation compared to 0 h (*n* = 3 biological replicates with 3 technical repeats each). (I) *Trem2* transcript level from RNA-seq data. (J) qPCR analysis of *Trem2* mRNA expression across different treatment groups (*n* = 3). (K and L) Western blot and analysis of TREM2, BAX, and BCL2 protein expression (*n* = 3). (*P < 0.05, **P < 0.01, ***P < 0.001, ****P < 0.0001; ns, not significant.)

To investigate the effects of radiation on macrophages, RAW264.7 cells were exposed to 5-Gy x-ray irradiation and harvested 24 h post-treatment for RNA-seq analysis. Our analysis identified 2,285 DEGs, comprising 1,549 up-regulated and 746 down-regulated genes (Fig. 3A). Subsequent GSEA revealed that *Trem2*-associated pathways were primarily enriched in myeloid cell activation and responses to external stimuli, with all of the top 10 terms showing significant up-regulation [normalized enrichment score (NES) > 1; Fig. 3B].

Integration of GO term analysis with RNA-seq data showed that the majority of genes were up-regulated following radiation exposure, with significantly altered DEGs displayed in Fig. 3C. Although changes in *Trem2* expression did not reach statistical significance, a modest up-regulation trend was observed (Log_2_FoldChange = 0.6). Collectively, these findings suggest that radiation modulates macrophage biology through multiple molecular pathways.

To validate these observations, we used flow cytometry to assess radiation-induced apoptosis. The apoptotic rate significantly increased following irradiation (Fig. 3D and E), consistent with our RNA-seq results showing up-regulation of proapoptotic *Nod2* and down-regulation of anti-apoptotic *Selenos* [34,35]. Furthermore, we observed decreased BCL2 expression accompanied by an increased BAX/BCL2 ratio (Fig. 3K and L), confirming radiation-triggered apoptosis through classical apoptotic pathways [36]. Complementary cell viability assays [integrated density (IntDent) and Cell Counting Kit-8 (CCK-8)] demonstrated decreased survival after irradiation (Fig. 3F to H), supporting earlier findings regarding cell viability after radiation [37,38].

Interestingly, our investigation of *Trem2* expression under radiation revealed discordance between mRNA and protein levels. Both RNA-seq and qPCR indicated transcriptional up-regulation of *Trem2*, whereas Western blotting showed decreased TREM2 protein abundance (Fig. 3K and L). This observation, along with our preliminary findings in skin tissue during early wound healing, suggests the existence of regulatory mechanisms controlling TREM2 protein expression in response to radiation.

### Radiation triggers the NRF2/ADAM17 axis to promote TREM2 shedding

**Fig. 4.**
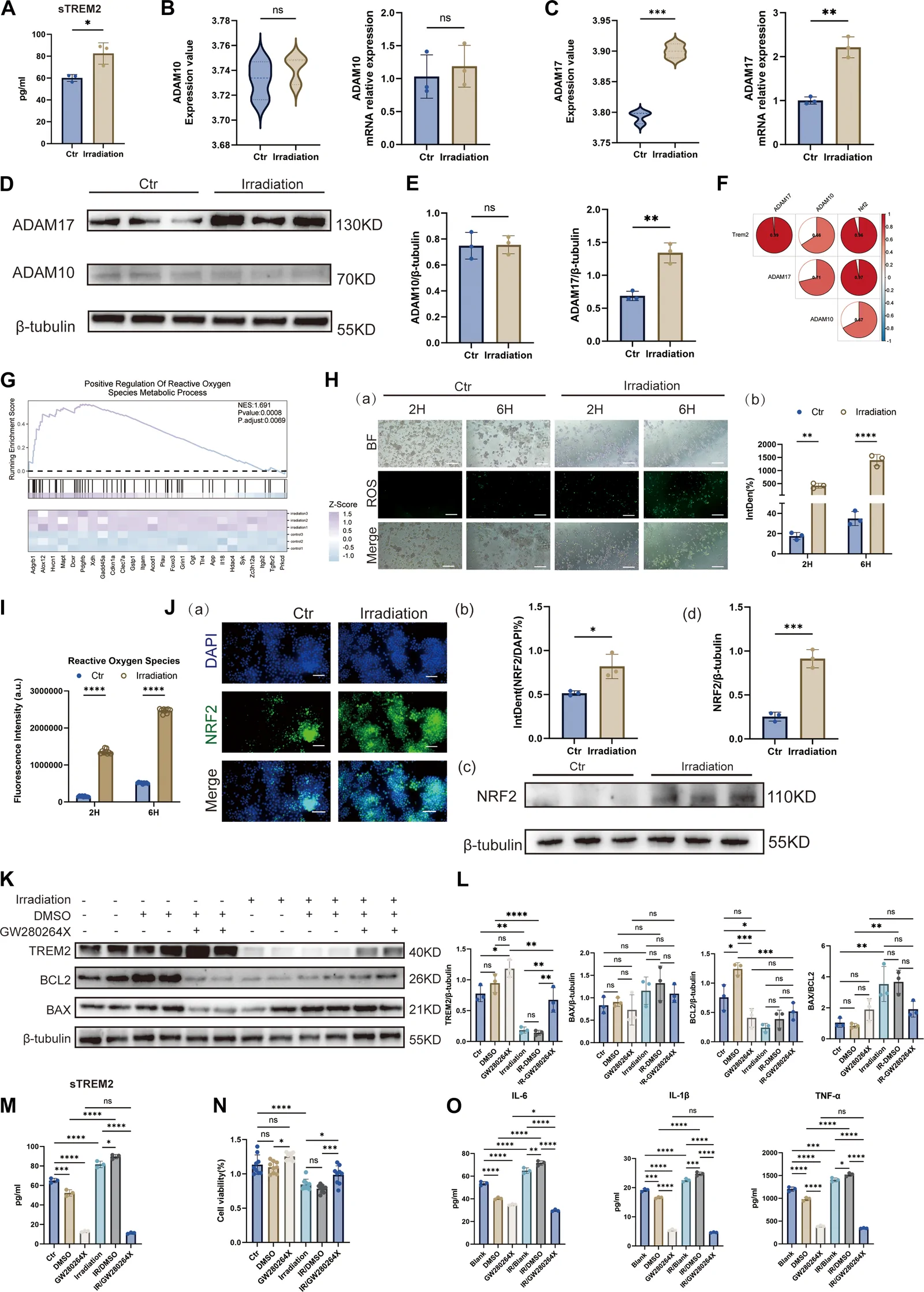
Radiation-induced ROS–Nrf2–ADAM17 axis promotes Trem2 shedding. (A) Quantification of sTREM2 in culture supernatants of RAW264.7 cells under control and irradiated conditions (*n* = 3). (B) ADAM10 transcript levels from RNA-seq and relative mRNA expression measured by qPCR (*n* = 3). (C) ADAM17 transcript levels from RNA-seq and relative mRNA expression measured by qPCR (*n* = 3). (D and E) Western blotting and analysis of ADAM10 and ADAM17 (*n* = 3). (F) Pearson correlation analysis between *Trem2*, *ADAM10*, *ADAM17*, and *Nrf2* based on RNA-seq data. (G) GSEA showing enrichment of the “Positive Regulation of Reactive Oxygen Species Metabolic Process” pathway after irradiation. (H) (a) ROS levels detected by fluorescence staining at 2 and 6 h post-irradiation (green), shown with brightfield (BF) (scale bar, 200 μm); (b) quantification of integrated fluorescence intensity (IntDen) (*n* = 3). (I) ROS quantification based on fluorescence intensity at excitation/emission wavelengths of 488/525 nm (*n* = 3). (J) (a and b) IF detection and quantification of NRF2 (green) with DAPI staining (*n* = 3; scale bar, 200 μm); (c and d) Western blotting and quantification of NRF2 (*n* = 3). (K and L) Western blotting and quantification of TREM2, BAX, and BCL2 protein levels in control, DMSO, and GW280264X cells with or without irradiation (*n* = 3). (M) ELISA detection of sTREM2 in various treatment groups (*n* = 3). (N) Cell viability assay using CCK-8 in control (Ctr), DMSO, and GW280264X (ADAM17/ADAM10 inhibitor) groups with or without irradiation (*n* = 3). (O) ELISA detection of proinflammatory cytokines IL-1β, TNF-α, and IL-6 in various treatment groups (*n* = 3). (*P < 0.05, **P < 0.01, ***P < 0.001, ****P < 0.0001; ns, not significant.)

TREM2 is known to associate with DAP12 through heterodimer formation, involving a specific interaction between a lysine residue in the transmembrane domain of DAP12 and an aspartic acid residue in the corresponding region of TREM2 [39]. Current evidence suggests that the proteolytic release of TREM2 from the cell membrane is primarily mediated by metalloproteinases, particularly ADAM10 and ADAM17 [40–42]. This cleavage releases sTREM2 from the plasma membrane. To determine whether radiation-induced TREM2 cleavage leads to reduced protein expression, we measured sTREM2 levels in cell supernatants using ELISA. As expected, sTREM2 levels increased after radiation exposure (Fig. 4A). Subsequent RNA-seq, qPCR, and Western blotting revealed that ADAM17, but not ADAM10, was up-regulated following irradiation (Fig. 4B to E). Recent research reported that ADAM17 is regulated by an antioxidant response element (ARE) and is dependent on NRF2 expression [43]. Using Pearson correlation analysis, we found a strong positive correlation between *Trem2*, ADAM17, and *Nrf2* (Fig. 4F).Given that radiation induces ROS accumulation and sustained oxidative stress [44,45], we performed GSEA and observed up-regulation of ROS metabolic pathways and related genes (Fig. 4G). To further investigate this, we measured ROS levels at 2 and 6 h post-irradiation, corresponding to the early response phase in macrophages. ROS levels were significantly elevated (Fig. 4H and I). Additionally, IF and Western blotting confirmed increased NRF2 expression (Fig. 4J).

To examine whether NRF2 directly regulates ADAM17 transcription, we analyzed NRF2 chromatin immunoprecipitation sequencing (ChIP-seq) datasets (GSE221843 [46]). Genome-wide profiling revealed NRF2 binding preferentially enriched around promoter regions, as shown by metagene analysis and peak distribution relative to transcription start sites (TSSs) (Fig. S8A to C). Notably, visualization of ChIP-seq tracks in Integrative Genomics Viewer (IGV) showed a prominent NRF2 peak located near the ADAM17 TSS (Fig. S8D), suggesting that ADAM17 may be a direct transcriptional target of NRF2. To experimentally validate this observation, we performed ChIP-qPCR assays in RAW264.7 cells. Chromatin was successfully sheared into 200- to 500-base pair fragments, and conventional PCR confirmed the presence of ADAM17 fragments specifically in NRF2 but not in immunoglobulin G (IgG) controls (Fig. S8E and F). Quantitative PCR further demonstrated significant enrichment of NRF2 with ADAM17 compared with IgG, and irradiation markedly increased NRF2 occupancy at this locus (Fig. S8G). These findings, together with previous studies [43,47], suggest that NRF2 not only plays a key role in defense against oxidative stress but also promotes ADAM17 expression. To verify whether ADAM17 mediates TREM2 shedding after irradiation, we first used the ADAM10/17 inhibitor GW280264X. ADAM17 inhibition restored TREM2 protein levels, which had been reduced by irradiation. Although *Trem2* mRNA levels showed minor fluctuations across groups, no significant changes were detected under irradiation, indicating that ADAM17 predominantly regulates TREM2 posttranslationally (Fig. 4K and L and Fig. S9A).

To further validate this relationship, we knocked down ADAM17 expression using small interfering RNA (siRNA). qPCR and Western blotting confirmed efficient ADAM17 silencing, which concomitantly increased TREM2 protein levels under irradiation but had no significant effects in control groups (Fig. S9B to F). Consistently, both pharmacological inhibition and genetic knockdown of ADAM17 reduced sTREM2 levels after irradiation (Fig. 4M and Fig. S9G), confirming the role of ADAM17 in TREM2 shedding. Next, we examined the functional impact of ADAM17 inhibition. GW280264X treatment increased cell viability in both control and irradiated groups, as assessed by CCK-8 assay (Fig. 4N), whereas apoptosis measured by the BAX/BCL2 ratio was not significantly altered (Fig. 4L and M). In contrast, si-ADAM17 not only increased cell viability (Fig. S9H) but also reduced radiation-induced apoptosis by flow cytometry (Fig. S9I and J). Moreover, si-ADAM17 altered macrophage polarization: The proportion of CD86^+^CD206^−^ M1 macrophages increased in controls but decreased under irradiation, whereas CD206^+^CD86^−^ M2 macrophages showed the opposite trend (Fig. S9K and L). These findings suggest that ADAM17-mediated TREM2 shedding promotes proinflammatory macrophage polarization in response to irradiation. We assessed the impact of ADAM17 inhibition on cytokine secretion. As expected, irradiation markedly increased IL-6, IL-1β, and TNF-α levels in blank and dimethyl sulfoxide (DMSO) groups (Fig. 4O). Treatment with GW280264X reduced expression of these cytokines under both basal and irradiated conditions, indicating a general anti-inflammatory effect. However, when comparing GW280264X-treated groups with or without irradiation, only IL-6 showed a significant decrease in the irradiated condition, whereas IL-1β and TNF-α levels were not significantly different. To further investigate the in vivo relevance, we administered GW280264X intraperitoneally for 5 consecutive days after irradiation and wounding. Wound area and closure rate were monitored up to 14 d, but no significant differences of wound closure rate and collagen deposition were observed between GW280264X-treated and control mice (Fig. S10A to E). ADAM17 inhibition successfully affected sTREM2 levels, which were significantly decreased on days 3 and 7 compared to the control group, indicating a reduction in TREM2 shedding (Fig S10F). Furthermore, the levels of proinflammatory cytokines, including IL-1β and TNF-α, were decreased, following a similar trend to the control group. Although IL-6 levels were also decreased compared to the control group, its suppression was delayed until day 7 following GW280264X treatment. Conversely, the anti-inflammatory cytokine IL-10 exhibited a decrease at day 14 (Fig S10G). Collectively, these results suggest that short-term pharmacological inhibition of ADAM17 alone is insufficient to accelerate wound repair in vivo.

In summary, our results demonstrate that radiation-induced ROS up-regulates NRF2, which in turn promotes ADAM17 expression. ADAM17 mediates TREM2 shedding, leading to enhanced apoptosis and proinflammatory macrophage polarization. Nevertheless, although systemic ADAM17 inhibition failed to significantly accelerate wound closure in vivo, our in vitro findings strongly validate this regulatory axis and highlight its potential as a therapeutic target.

### TREM2 improves radiation resistance of macrophages by reducing apoptosis

**Fig. 5.**
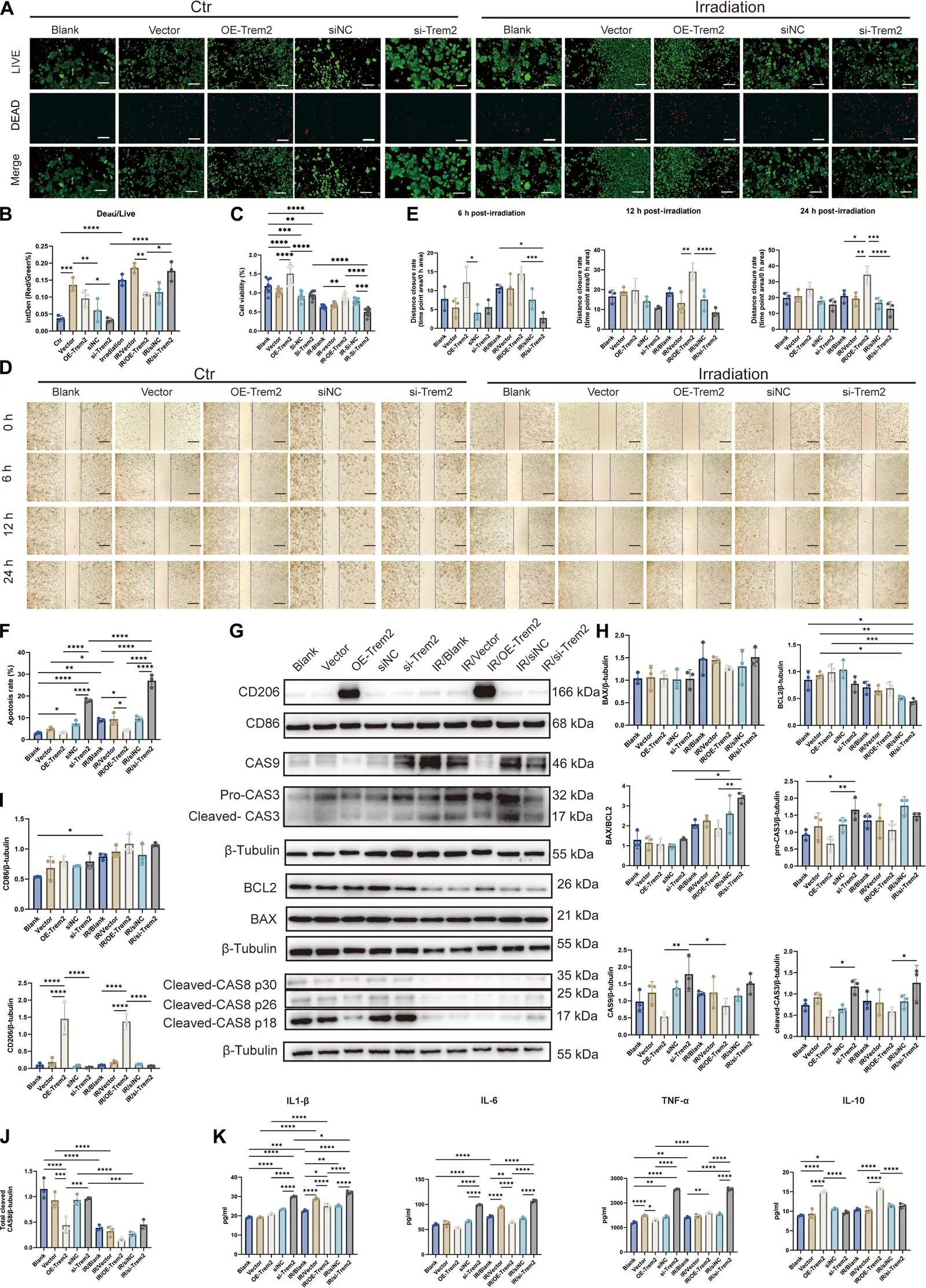
*Trem2* overexpression improves radiation resistance of RAW264.7 cells by reducing apoptosis. (A and B) Live/dead staining using Calcein-AM (live, green) and EthD-1 (dead, red); quantification of dead cells based on integrated fluorescence intensity (*n* = 3; scale bar, 200 μm). (C) Cell viability assessed by CCK-8 assay in Blank, Vector, OE-Trem2, siNC, and si-Trem2 groups with or without irradiation (*n* = 3). (D and E) Scratch assay and distance closure rate quantified at 6, 12, and 24 h relative to 0 h (*n* = 3; scale bar, 200 μm). (F) Apoptosis rates determined by flow cytometry across different treatment groups (*n* = 3). (G to J) Western blotting and analysis of CD206, CD86, CASPASE-9 (CAS9), total and cleaved CASPASE-3 (pro-CAS3, cleaved CAS3), BCL2, BAX, and cleaved CASPASE-8 (cleaved CAS8) (*n* = 3). (K) ELISA analysis of proinflammatory cytokines (IL-1β, TNF-α, and IL-6) and the anti-inflammatory cytokine IL-10 in different groups (*n* = 3). (*P < 0.05, **P < 0.01, ***P < 0.001, ****P < 0.0001; only statistically significant comparisons are shown in panel.)

To further investigate the role of TREM2 under radiation conditions, we established *Trem2*-overexpressing (OE-*Trem2*) and *Trem2*-knockdown (si-*Trem2*) RAW264.7 macrophages. Successful construction was confirmed using qPCR, Western blotting, and sTREM2 detection (Fig. S11A to E). Initial viability assessments using live/dead staining revealed that, although OE-*Trem2* cells exhibited a nonsignificant reduction in cell death compared with vector controls under normal conditions, si-*Trem2* cells showed significantly decreased cell death relative to OE-*Trem2*. Remarkably, this pattern was reversed under radiation exposure, where OE-*Trem2* cells demonstrated significantly enhanced survival compared with both vector and si-*Trem2* groups (Fig. 5A and B). These findings were corroborated by CCK-8 assays, which consistently showed improved viability of OE-*Trem2* cells regardless of radiation exposure, whereas si-*Trem2* exhibited the opposite effect (Fig. 5C). Subsequent scratch assays assessing proliferation dynamics demonstrated that OE-*Trem2* cells accelerated wound closure, while si-*Trem2* cells failed to show closure at 6 h post-scratch under both irradiated and nonirradiated conditions. Although no significant differences were observed at 12 and 24 h under normal conditions, OE-*Trem2* cells exhibited significantly enhanced proliferation following radiation exposure at later time points (Fig. 5D and E). Flow cytometry analysis revealed that si-*Trem2* not only induced apoptosis in control cells but also exacerbated radiation-induced cell death, whereas OE-*Trem2* effectively mitigated these effects (Fig. S11F and Fig. 5F). Further characterization of macrophage polarization showed that OE-*Trem2* promoted an M2 phenotype (evidenced by increased CD206 expression) while exerting minimal influence on M1 markers (CD86) (Fig. 5G and H). We then evaluated BAX, BCL2, and CASPASE9 (indicating the intrinsic apoptosis pathway), CASPASE8 (indicating the extrinsic pathway), and the effector CASPASE3. Notably, the BAX/BCL2 ratio increased under radiation conditions, consistent with previous findings. The combined results of CASPASE9, cleaved CASPASE3, and the BAX/BCL2 ratio showed that CASPASE9 activation correlated with the up-regulation of BAX/BCL2, indicating mitochondrial outer membrane permeabilization as a key event. Cleaved CASPASE3 levels were significantly increased in the si-*Trem2* group after irradiation, consistent with elevated CASPASE9 and BAX/BCL2 ratios, confirming enhanced execution-phase apoptosis when *Trem2* was silenced under radiation conditions. In contrast, OE-*Trem2* attenuated CASPASE3 activation, suggesting a protective role through inhibition of the caspase cascade. Furthermore, CASPASE8 expression was generally reduced in radiation-treated groups relative to controls, indicating the possible involvement of alternative apoptotic pathways under radiation. However, OE-*Trem2* further decreased cleaved CASPASE8 levels, whereas si-*Trem2* did not restore or enhance its activation, suggesting that TREM2 may also modulate extrinsic apoptotic signaling (Fig. 5G to J).

Finally, cytokine profiling demonstrated that OE-*Trem2* reduced proinflammatory factors (TNF-α, IL-6, and IL-1β) while increasing the anti-inflammatory cytokine IL-10, potentially through its promotion of M2 polarization (Fig. 5K).

### TREM2 deficiency exacerbates radiation-induced apoptosis via suppression of ERK signaling

**Fig. 6.**
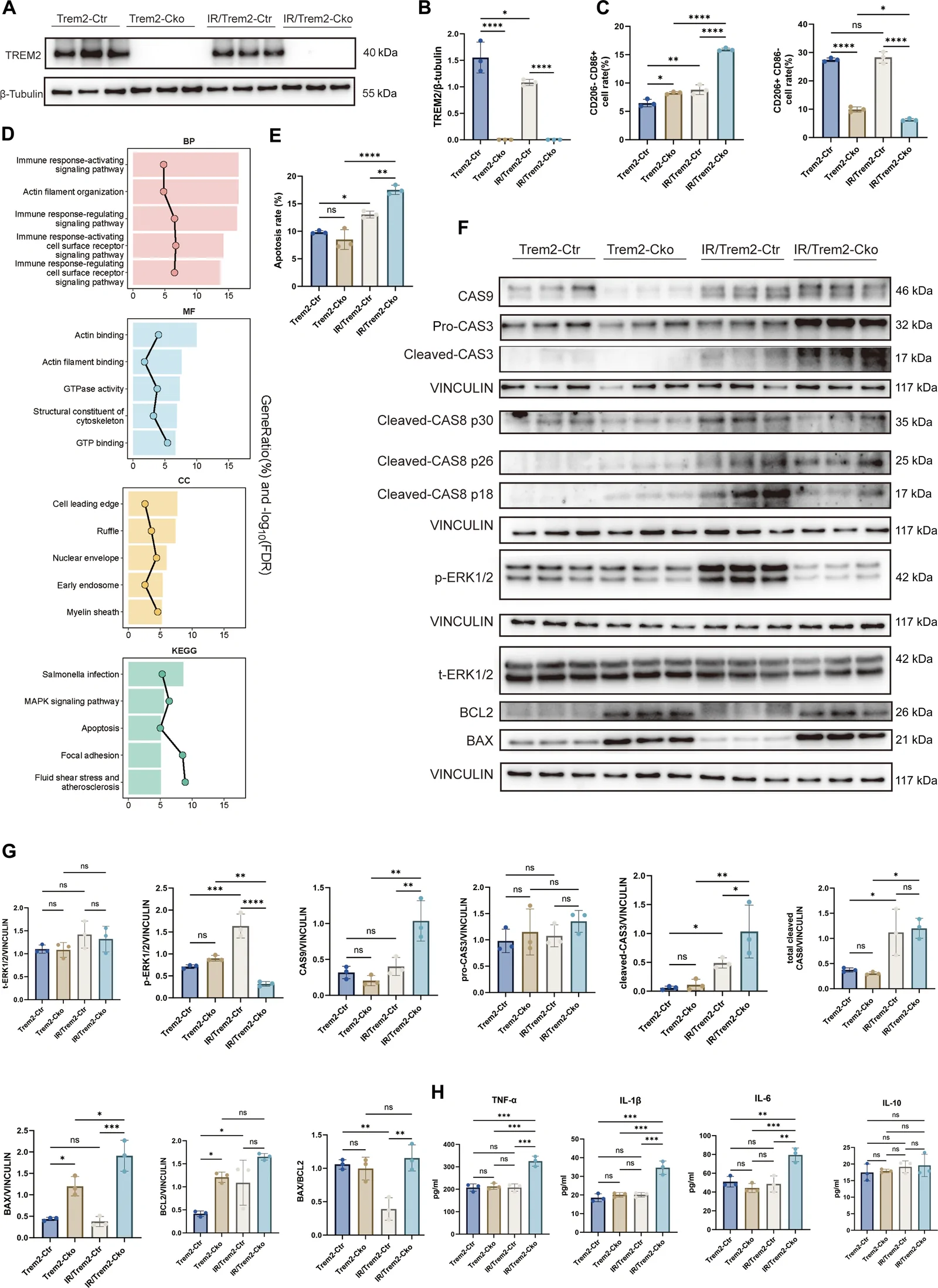
Trem2 deficiency exacerbates radiation-induced apoptosis by suppressing ERK phosphorylation. (A and B) Western blotting and quantification of TREM2 expression in *LysM*^Cr*e*^*Trem2*^flox/flox^ (Trem2-Cko) and *LysM*^−^*Trem2*^flox/flox^ (Trem2-Ctr) BMDM, with or without irradiation (*n* = 3). (C) Flow cytometry analysis of macrophage polarization based on M1 markers (CD86^+^CD206^−^) and M2 markers (CD86^−^CD206^+^) (*n* = 3). (D) Gene Ontology (GO) and Kyoto Encyclopedia of Genes and Genomes (KEGG) pathway enrichment analysis based on RNA-seq data. (E) Apoptosis rate assessed by Annexin V/PI flow cytometry in Trem2-Ctr and Trem2-Cko macrophages after irradiation (*n* = 3). (F and G) Western blotting and analysis of phosphorylated ERK1/2 (p-ERK), total ERK1/2 (t-ERK), CASPASE-9 (CAS9), total and cleaved CASPASE-3 (pro-CAS3, cleaved CAS3), BCL2, BAX, and cleaved CASPASE-8 (cleaved CAS8) (*n* = 3). (H) ELISA quantification of proinflammatory cytokines (IL-1β, TNF-α, and IL-6) and anti-inflammatory cytokine IL-10 in Trem2-Ctr and Trem2-Cko groups (*n* = 3). (*P < 0.05, **P < 0.01, ***P < 0.001, ****P < 0.0001; ns, not significant.)

We used bone marrow-derived macrophages (BMDMs) from *LysM*^Cr*e*^*Trem2*^flox/flox^ (Trem2-Cko) and LysM^−^*Trem2*^flox/flox^ (Trem2-Ctr) mice to investigate the effects of *Trem2* knockout under irradiation (Fig. S12A). PCR analysis of *Trem2*^flox/flox^ and *LysM*^Cre/−^ cells, along with qPCR of *Trem2*, confirmed successful gene deletion (Fig. S12B and C). Notably, *Trem2* expression was down-regulated in Trem2-Ctr BMDMs following irradiation, consistent with our previous findings in RAW264.7 cells. As expected, *Trem2* was undetectable in Trem2-Cko BMDMs regardless of radiation exposure (Fig. 6A and B). Radiation exposure induced a shift toward M1-type macrophage polarization in BMDMs, mirroring the polarization pattern observed in RAW264.7 cells. Flow cytometric analysis using CD86 and CD206 markers revealed that *Trem2* deficiency enhanced M1 polarization while suppressing M2 polarization (Fig. S12D and Fig. 6C). To explore the transcriptional consequences of *Trem2* deletion, we performed RNA-seq on Trem2-Ctr and Trem2-Cko BMDMs. Differential expression analysis identified 117 up-regulated and 197 down-regulated genes in Trem2-Cko cells (Fig. S13A). Integration with the publicly available dataset CRA01171145 from the BIG Data Center identified 367 hub genes associated with *Trem2* deficiency and irradiation. GO and Kyoto Encyclopedia of Genes and Genomes (KEGG) pathway enrichment analyses highlighted the MAPK signaling pathway, particularly the extracellular signal-regulated kinase 1/2 (ERK1/2) cascade (Fig. S13B to D). Further, GO and KEGG analyses of genes highly correlated with *Trem2* (Pearson’s *r* > 0.8) reaffirmed the association between MAPK signaling and apoptotic pathways (Fig. 6D). To validate these findings, apoptosis was evaluated using flow cytometry. Consistent with RAW264.7 results, radiation markedly increased apoptosis in BMDMs, with the irradiated Trem2-Cko group showing the highest apoptotic rate. Minimal apoptosis was observed under nonirradiated conditions, with no significant difference between Trem2-Ctr and Trem2-Cko cells (Fig. S12E and Fig. 6E). Western blot analysis confirmed the molecular signature of increased apoptosis. Among irradiated BMDMs, the Trem2-Cko group showed a significantly higher BAX/BCL2 ratio than Trem2-Ctr, indicating a shift toward proapoptotic signaling. This ratio remained unchanged in nonirradiated Trem2-Cko cells. Furthermore, cleaved CASPASE3 levels increased after irradiation, and TREM2 deficiency further amplified this effect. CASPASE9 expression was also significantly elevated in irradiated Trem2-Cko cells. Consistently, JC-1 staining revealed a marked loss of mitochondrial membrane potential (Δψm) in irradiated Trem2-Cko macrophages compared with Trem2-Ctr cells (Fig. S12H and I), confirming mitochondrial dysfunction and indicating that *Trem2* deficiency exacerbates intrinsic apoptotic activation. Total cleaved CASPASE8 levels were elevated in both Trem2-Ctr and Trem2-Cko cells following irradiation, potentially explaining the increase in cleaved CASPASE3 in Trem2-Ctr cells despite limited CASPASE9 activation and a decreased BAX/BCL2 ratio (Fig. 6F and G). To explore the signaling mechanism consistent with the RNA-seq results, we next examined the ERK pathway. Although total ERK levels remained constant, phosphorylated ERK (p-ERK) was robustly induced by radiation in Trem2-Ctr cells but was significantly reduced in Trem2-Cko cells (Fig. 6F and G), suggesting that TREM2 is required for ERK activation under irradiation stress.

To further establish causality, we performed rescue experiments using the ERK1/2 agonist Ro 677476, a potent activator of ERK phosphorylation. Treatment of Trem2-Cko BMDMs with Ro 677476 significantly restored ERK1/2 phosphorylation (Fig. S14A and B), reduced irradiation-induced apoptosis (Fig. S14C and D), and increased cell viability as assessed by CCK-8 assay (Fig. S14E). Moreover, ERK agonist treatment improved mitochondrial function and reduced active CASPASE3 fluorescence, confirming that ERK activation alleviates irradiation-induced apoptosis in *Trem2*-deficient BMDMs (Fig. S14F to H). Consistently, *Trem2* overexpression in RAW264.7 cells markedly increased p-ERK1/2 levels compared with si-*Trem2* and control groups (Fig. S14I and J).

Collectively, these findings establish ERK as a downstream mediator of TREM2 in maintaining mitochondrial integrity and suppressing apoptosis under irradiation stress. Lastly, proinflammatory cytokines TNF-α, IL-6, and IL-1β were significantly up-regulated in irradiated Trem2-Cko BMDMs, whereas other groups showed no significant changes. IL-10 expression remained unchanged across all conditions, indicating that TREM2 deficiency potentiates radiation-induced inflammation (Fig. 6H).

### TREM2 deficiency increases inflammation and impairs radiation wound healing

**Fig. 7.**
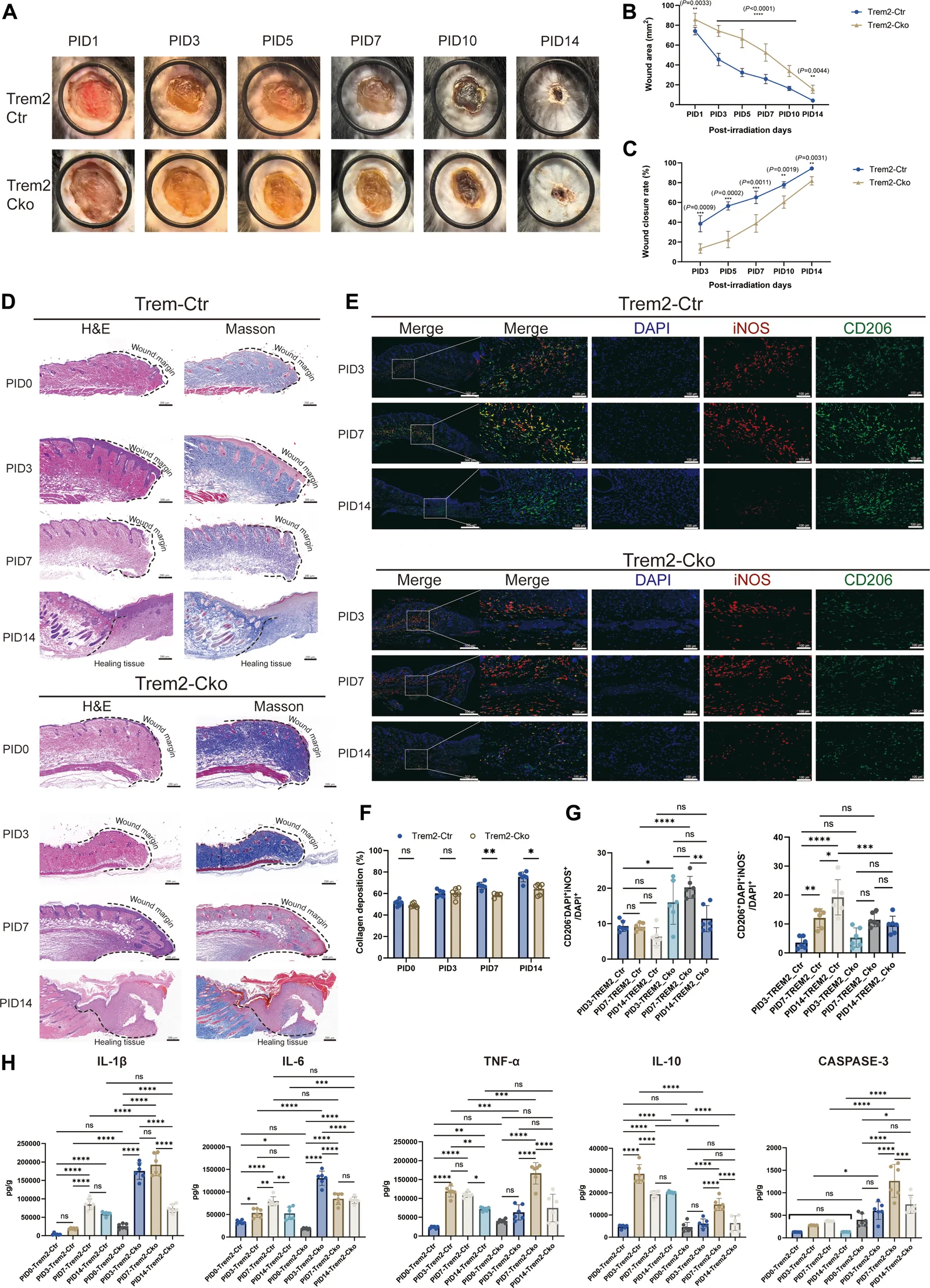
Trem2 deficiency impairs wound healing in RISI. (A) RISI mouse model: Trem2-Ctr and Trem2-Cko mice were subjected to 5-Gy total body x-ray irradiation followed by full-thickness excisional wounding (black circle, 12-mm inner diameter). (B) Representative images of wound areas in each group (*n* = 6). (C) Quantification of wound closure rates at indicated time points relative to PID0 (*n* = 6). (D) H&E and Masson staining of wound tissues at PID0, 3, 7, and 14 (*n* = 6). (E) IF costaining of iNOS (red), CD206 (green), and DAPI (blue) to identify M1- or M2-type macrophages in Trem2-Ctr and Trem2-Cko groups (scale bars shown in panels). (F) Collagen deposition quantified from Masson staining (*n* = 6). (G) Quantification of macrophage subsets, calculated as the proportion of iNOS^+^CD206^−^DAPI^+^ cells (M1) or iNOS^−^CD206^+^DAPI^+^ cells (M2) among total DAPI^+^ cells (*n* = 6). (H) ELISA quantification of proinflammatory cytokines (IL-1β, TNF-α, and IL-6), anti-inflammatory cytokine IL-10, and total CASPASE3 in Trem2-Ctr and Trem2-Cko groups (*n* = 6).

Finally, to assess the effects of *Trem2* deficiency in vivo, Trem2-Ctr and Trem2-Cko mice were subjected to 5-Gy x-ray radiation, followed by artificial wound creation. *Trem2* knockout led to significantly delayed wound healing under irradiation, as evidenced by marked differences in wound size and healing rate (Fig. 7A to C). Consistent with these observations, H&E and Masson staining revealed reduced collagen deposition in Trem2-Cko mice (Fig. 7D and F). To further investigate macrophage polarization, we performed inducible nitric oxide synthase (iNOS) and CD206 IF staining. Under irradiation, *Trem2* deficiency increased the proportion of CD206^−^DAPI^+^iNOS^+^ M1 macrophages at days 3 and 7, with no significant difference at day 14. Conversely, the proportion of CD206^+^DAPI^+^iNOS^−^ M2 macrophages was consistently reduced at all time points (Fig. 7E and G), consistent with our in vitro results. Collectively, these findings indicate that TREM2 deficiency sustains a proinflammatory macrophage phenotype during the early inflammatory phase while impairing the induction of anti-inflammatory and reparative macrophages during the healing phase, ultimately leading to defective wound repair in RISI. To evaluate the inflammatory microenvironment, cytokine levels in adjacent wound tissues were measured by ELISA. In Trem2-Ctr mice, TNF-α levels peaked at day 3 and declined significantly by days 7 and 14. IL-6 and IL-1β peaked at day 7 before decreasing, while IL-10 peaked at day 7 and remained relatively stable through day 14. In contrast, Trem2-Cko mice exhibited a distinct cytokine profile: TNF-α expression was significantly higher than Trem2-Ctr at day 3 and remained elevated through day 7 before declining by day 14. IL-6 peaked earlier (day 3), remained high through day 7, and declined only modestly by day 14. IL-1β also showed an earlier and stronger induction, rising from day 3 and reaching a higher peak at day 7 compared with Trem2-Ctr. Notably, IL-10 levels increased at day 7 but dropped abruptly by day 14, contrasting with the sustained expression in Trem2-Ctr mice. Comparative analysis confirmed that proinflammatory cytokines (TNF-α, IL-6, and IL-1β) were significantly higher in Trem2-Cko mice than in Trem2-Ctr mice at most time points, whereas the anti-inflammatory cytokine IL-10 was markedly reduced after its transient peak (Fig. 7H). In addition, apoptosis was evaluated by measuring total CASPASE3 levels via ELISA. Trem2-Cko mice displayed significantly increased CASPASE3 expression, particularly at day 7, compared with Trem2-Ctr groups (Fig. 7H).

To further assess the functional role of TREM2^+^ macrophages, we isolated BMDMs and separated TREM2^+^ and TREM2^−^ subsets by flow cytometry (Fig. S15A). These sorted cells were locally injected into irradiated wounds. Notably, TREM2^+^ macrophage transfer significantly accelerated wound closure and collagen deposition compared with TREM2^−^ macrophages (Fig. S15B to F). Strem2 was elevated in each time point, which indicated the successful local transplantation (Fig. S15G). Mechanically, all proinflammatory cytokines ((TNF-α, IL-6, and IL-1β) were decreased and high expression of anti-inflammatory cytokine IL-10 was detected (Fig. S15H), underscoring the protective role of TREM2^+^ macrophages in promoting tissue repair.

Taken together, these data demonstrate that TREM2 deficiency disrupts the normal inflammatory resolution process, leading to sustained proinflammatory cytokine production, insufficient anti-inflammatory signaling, and enhanced apoptosis, thereby impairing the healing of radiation-induced wounds. Conversely, local replenishment of *Trem2^+^* macrophages effectively ameliorated these defects and promoted wound repair, highlighting their therapeutic potential in RISI.

## Discussion

RISI remains a significant clinical complication, particularly among cancer patients undergoing radiotherapy, where the skin is often the primary site of exposure [8,48]. Macrophages are critical regulators of the wound microenvironment, and their dysfunction under radiation stress contributes substantially to chronic inflammation and impaired tissue regeneration [32]. However, the molecular regulators orchestrating macrophage fate during RISI remain incompletely understood. In this study, we comprehensively investigated the role of *Trem2* subsets in macrophage-mediated responses to RISI by combining single-cell transcriptomics, molecular analyses, and in vivo wound-healing assessments. Our findings identified TREM2 as a regulator of macrophage survival and polarization following irradiation.

Accumulating evidence suggests that *Trem2^+^* macrophages represent a specialized regulatory subset particularly relevant in fibrosis, neuroinflammation, and metabolic diseases [42,49,50]. Previous studies have highlighted the anti-apoptotic and immunomodulatory functions of TREM2, especially in neurodegenerative and cardiac diseases [22,50–52]; however, its role in radiation-induced apoptotic signaling has not been clearly delineated. Here, our scRNA-seq analysis revealed a distinct *Trem2^+^* macrophage subset induced after irradiation. According to CellChat analysis, *Trem2^+^* macrophages exhibited more frequent and stronger interactions with other cell clusters, particularly T cells and Th17 cells, through several proinflammatory and chemotactic ligand–receptor pairs [53,54]. These interactions are known to regulate T cell recruitment, activation, and positioning within injured tissues [55,56], highlighting a potentially unique immunoregulatory role of *Trem2* in macrophage–T cell crosstalk. Moreover, prominent autocrine signaling within *Trem2^+^* macrophages suggest a self-sustaining loop of activation or survival signaling that may amplify their functional output. To ensure that the emergence of *Trem2^+^* macrophages was not model-specific, we compared their behavior across different irradiation contexts. For scRNA-seq, 15-Gy irradiation was used to maximize immune perturbations and capture sufficient cellular heterogeneity [57,58]. For subsequent wound-healing experiments, we employed a 5-Gy model, a sublethal dose widely used in preclinical combined-injury research to mimic clinically relevant conditions while preserving animal viability [59–62]. Importantly, *Trem2* expression and the enrichment of *Trem2^+^* macrophages were also validated in the 5-Gy model, confirming that the subset identified at 15 Gy reflects a conserved macrophage response across irradiation conditions.

Our findings further identified a regulatory mechanism by which radiation impairs TREM2 signaling through enhanced proteolytic shedding. We observed radiation-induced increases in sTREM2, accompanied by decreased cellular TREM2 protein levels despite transcriptional up-regulation. This discrepancy was mechanistically linked to the ROS–NRF2–ADAM17 axis: radiation elevated intracellular ROS levels, induced NRF2 expression, and up-regulated ADAM17, a sheddase known to cleave TREM2 [40,43]. Consistent with this mechanism, siRNA-mediated knockdown of *Adam17* preserved TREM2 protein levels under irradiation and reduced sTREM2 release, consistent with pharmacological inhibition using GW280264X. These findings confirm the central role of ADAM17 in TREM2 shedding under irradiation. Functionally, *si-Adam17* improved macrophage survival and shifted polarization, reducing proinflammatory M1 macrophages and enhancing M2 polarization after irradiation. Pharmacological inhibition of ADAM17 yielded a partially overlapping but distinct cytokine profile: GW280264X broadly suppressed cytokine secretion under both basal and irradiated conditions. However, only IL-6 levels were significantly decreased under irradiation, whereas IL-1β and TNF-α remained unchanged. Previous studies have shown that ADAM17 is the principal sheddase responsible for IL-6Rα cleavage during inflammation, and that loss of ADAM17 markedly diminishes soluble IL-6R levels and IL-6 trans-signaling [63,64]. Thus, ADAM17 inhibition may not only limit IL-6R shedding-dependent trans-signaling but also attenuate nuclear factor κB (NF-κB)- and STAT3-driven IL-6 production [41,65]. Consequently, the pronounced reduction of IL-6 in our model likely reflects both impaired trans-signaling and suppression of upstream inflammatory cascades. Nevertheless, in vivo administration of GW280264X for 5 consecutive days post-irradiation did not significantly accelerate wound closure. This discrepancy may be due to pharmacokinetic limitations, insufficient local drug accumulation, or the pleiotropic roles of ADAM17 as a sheddase for multiple substrates beyond TREM2 [65,66]. These results highlight the complexity of targeting ADAM17 in vivo and suggest that localized inhibition or more selective strategies may be required for therapeutic efficacy. Importantly, ADAM17-mediated TREM2 shedding and sTREM2 release were most evident during the early post-irradiation phase, implicating a role in initiating the inflammatory cascade.

Previous work has shown that sTREM2 can exert functional effects in other contexts: In Alzheimer’s disease, exogenous sTREM2 enhanced microglial survival, clustering, and Aβ clearance [67], while in human monocyte-derived macrophages, sTREM2 dynamically modulated cytokine production—initially promoting proinflammatory mediators and later supporting anti-inflammatory responses [68,69]. The elevated sTREM2 observed here supports irradiation-induced TREM2 shedding via the ROS–NRF2–ADAM17 axis. Although we did not directly test sTREM2 function, prior findings suggest that it regulates survival and cytokine responses in myeloid cells. Thus, sTREM2 may serve as a biomarker of irradiation stress and a potential modulator of immune responses, an avenue warranting further investigation. Overall, our findings uncover a novel mechanism of TREM2 regulation under oxidative stress, which may compromise its protective functions in irradiated macrophages by limiting cell-surface receptor availability and impairing DAP12-associated signaling [70].

Interestingly, we observed divergent apoptotic responses between RAW264.7 macrophages and bone marrow-derived macrophages (BMDMs). In RAW264.7 cells, irradiation reduced CASPASE8 levels while increasing the BAX/BCL2 ratio and activating CASPASE9, consistent with mitochondria-dependent intrinsic apoptosis [36]. In contrast, BMDMs exhibited elevated CASPASE8 expression and stable BAX/BCL2 ratios, suggesting a preference for receptor-mediated extrinsic apoptosis [36]. These differences likely reflect cell type-specific regulatory mechanisms: Primary BMDMs retain intact death receptor [71] pathways, whereas immortalized RAW264.7 cells may rely more heavily on intrinsic apoptosis due to altered signaling [72–74].

In parallel, our results confirm the crucial role of TREM2 in macrophage polarization. Prior studies have established that TREM2 promotes M2-like anti-inflammatory phenotypes while limiting M1 responses [75]. Consistent with this, TREM2 deficiency in both RAW264.7 cells and BMDMs enhanced M1 markers (CD86, TNF-α, and IL-6) and suppressed M2 markers (CD206 and IL-10), particularly under irradiation. Mechanistically, TREM2 mediated its protective effects partly through the ERK signaling pathway. ERK activation was significantly reduced in TREM2-deficient macrophages following irradiation, coinciding with increased apoptosis, elevated proinflammatory cytokines, and reduced M2 polarization. These findings align with previous reports that ERK signaling promotes cell survival intrinsic apoptotic pathway and anti-inflammatory macrophage phenotypes [76,77]. Thus, the TREM2–ERK axis may serve as a central regulatory module governing macrophage fate under radiation stress, balancing cell survival and apoptosis.

In vivo, TREM2 deficiency led to sustained inflammation, reduced collagen deposition, and delayed wound healing, reinforcing its role in orchestrating macrophage-mediated tissue repair. Notably, adoptive transfer experiments demonstrated that local injection of flow cytometry-sorted TREM2^+^ macrophages into irradiated wounds significantly accelerated wound closure compared with TREM2^−^ macrophages. These results underscore the therapeutic relevance of TREM2^+^ macrophages, consistent with previous studies showing that they enhance tissue regeneration in muscle and liver by promoting phagocytosis, anti-inflammatory polarization, and extracellular matrix remodeling [17,25,75]. Together, these findings suggest that local supplementation of TREM2^+^ macrophages represents a promising strategy for mitigating RISI.

Despite these insights, this study has several limitations. We did not explore alternative forms of programmed cell death, such as necroptosis, pyroptosis, or ferroptosis, which may also contribute to macrophage loss during RISI [38]. Although sTREM2 levels increased after irradiation, its biological roles in the wound microenvironment remain undefined. Furthermore, while our results highlight ERK signaling as a key downstream pathway of TREM2, potential non-ERK cascades warrant further investigation. Lastly, the use of different radiation doses across scRNA-seq, bulk RNA-seq, and in vivo models may introduce variability, although the convergent results suggest that the findings are robust.

**Fig. 8.**
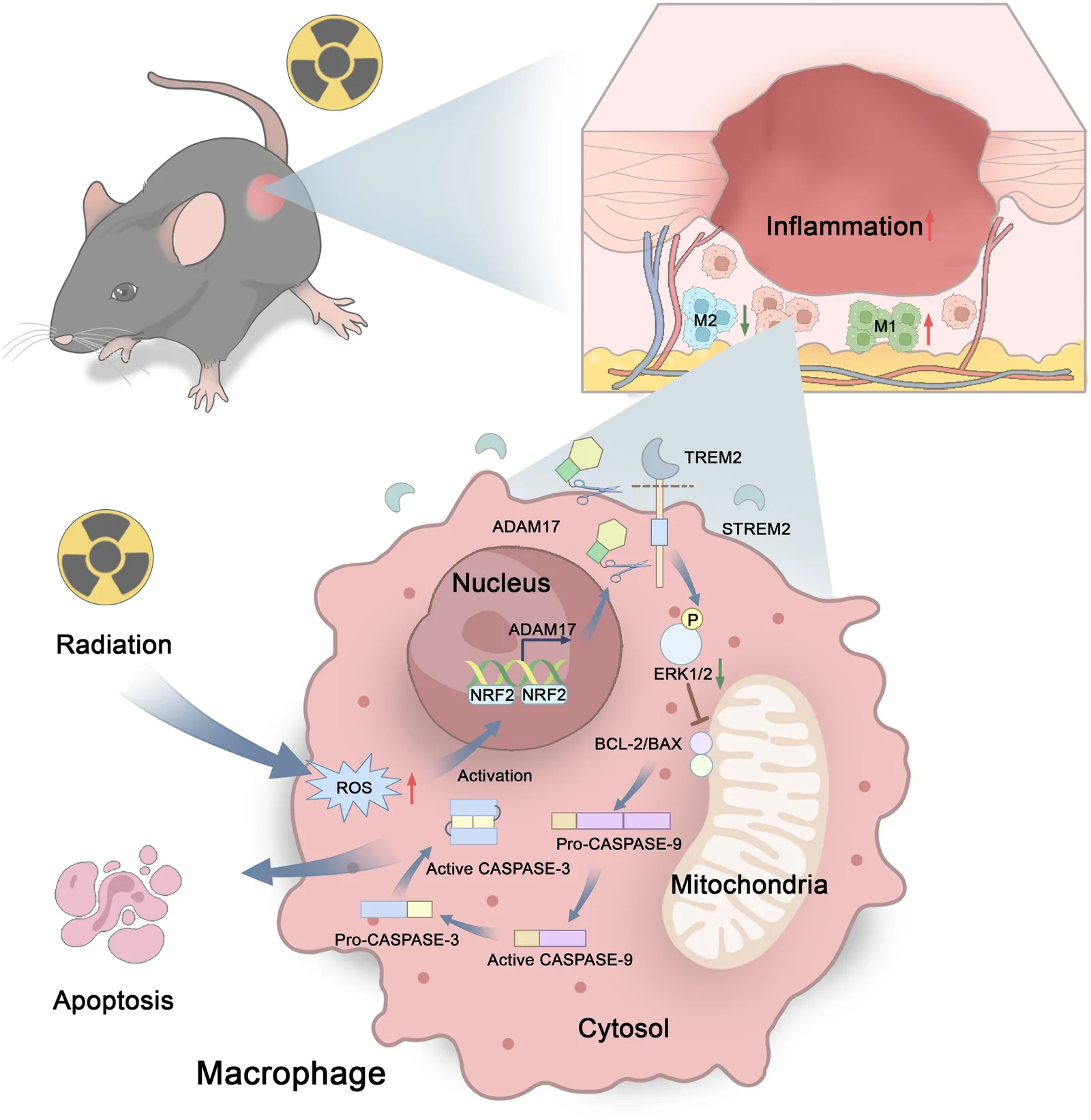
Schematic of TREM2 regulates macrophage apoptosis and impairs wound healing following RISI.

Collectively, our results provide new mechanistic insights into macrophage dysfunction during RISI. Specifically, we demonstrate that radiation-induced oxidative stress activates the ROS–NRF2–ADAM17 axis, leading to TREM2 shedding and impaired receptor availability, and that TREM2 regulates macrophage survival through the ERK pathway. These findings suggest that targeting the ROS–NRF2–ADAM17–TREM2–ERK signaling cascade may offer therapeutic opportunities for mitigating radiation-induced tissue damage and promoting wound healing (Fig. 8).

Radiation induces excessive ROS accumulation, which activates the NRF2–ADAM17 axis, promoting the shedding of TREM2. Reduced TREM2 impairs downstream ERK1/2 phosphorylation, leading to enhanced macrophage apoptosis and dysregulated polarization. Specifically, TREM2 deficiency suppresses M2-like polarization while promoting mitochondria-dependent intrinsic apoptosis, ultimately disrupting macrophage survival, resolution of inflammation, and collagen deposition. In contrast, TREM2 overexpression enhances cell viability and skews macrophage polarization toward an anti-inflammatory M2 phenotype. Together, these findings identify TREM2 as a key protective regulator in macrophage-mediated wound healing under radiation-induced stress.

## Materials and Methods

### scRNA-seq analysis

We used the dataset GSE201447 [10] to obtain radiation-induced macrophage subsets. Seurat (version 5.0.1) [78] in R (version 4.3.0) was used for downstream analysis. To obtain high-quality cell data, we set 200 to 2500 detected genes and less than 5% mitochondrial genes as the threshold to filter the raw data, followed by normalization of the filtered data, and used the *t*-distributed stochastic neighbor embedding (tSNE) as the re-education size. Cell clustering was completed using Wilcoxon rank sum tests through the “FindAllmakers” function, followed by cell annotation using CellMarker 2.0 [79], and cell interaction analysis was performed by CellChat[80]. The irGSEA package (version 3.2.7) [81] was employed for GSEA, AUCell, UCell scoring, and GO analyses, and a pseudotemporal analysis was performed using the Monocle package [82].

### Animals and radiation

All animal procedures were approved by the Animal Care and Use Committee of Zhongshan Hospital, affiliated with Fudan University for Animal Welfare (approval no. 2024–151). All experiments were conducted in compliance with the Guide for the Care and Use of Laboratory Animals. Male C57BL/6JGpt mice aged 6 to 8 weeks, used as the WT model, were purchased from GemPharmatech (Nanjing, China). TREM2^flox/flox^ mice (Shanghai Model Organisms) were crossbred with LysM^Cre^ mice (Nan Jing Xietong Biotechnology Company) to generate LysM^Cre^TREM2^flox/flox^ (Trem2-Cko) mice. TREM2^flox/flox^ littermates served as controls (Trem2-Ctr). All mice were maintained on the C57BL/6 background. For in vivo studies, mice were randomly assigned into groups (*n* = 18 per group). To create a RISI model, mice were subjected to a total of 5 Gy of whole-body irradiation at a 320-kV, 12.5-mA, 1 Gy/min dose rate under an x-ray irradiator (x-RAD 320, Precision X-RAY, Madison, USA). After irradiation, dorsal hair was shaved, and a depilatory cream was applied. Then, a 10-mm full-thickness cutaneous wound was created on the dorsal midline of the mice that were placed under anesthesia through intraperitoneal injection of 0.3% phenobarbital sodium (0.1 ml/10 g). Wound healing was monitored over a period of 14 d. On days 1, 3, 7, 10, and 14, photographic images of the wounds were obtained and used to analyze the wound closure rate. Adjacent wound tissues were harvested in days 0, 3, 7, and 14 for subsequent molecular and histological studies.

### Histology

Skin samples were fixed using 4% paraformaldehyde, embedded in paraffin, and cut into 4-μm-thick transverse sections. For histological evaluation, tissue sections were deparaffinized and rehydrated, followed by H&E and Masson’s trichrome staining. Image analysis was conducted using ImageJ software. For immunohistochemistry staining, the sections were incubated with primary anti-TREM2 (1:500, ab305103, Abcam, Cambridge, UK), anti-F4/80 (1:600, 70076, Cell Signaling Technology, MA, USA), anti-iNOS (1:1,000, ab3523, Abcam, Cambridge, UK), and anti-CD206 (1:500, HA722892, Huabio, Hangzhou, China) antibodies overnight at 4 °C after blocking endogenous peroxidase activity. After incubation, a Multiplex IHC kit (WAS1804, WASci Biotechnology, Shanghai, China) was used for detection according to the manufacture’s instruction. All slides were scanned using a 3DHISTECH Pannoramic 250 Flash III scanner (3DHISTECH, Budapest, Hungary).

### Cell culture and radiation

RAW264.7 cells were purchased from Pricella Biotechnology (CL-0190, Wuhan, China) and cultured in RAW264.7 Cell Complete Medium (CM-0190, Pricella Biotechnology, Wuhan, China). Murine bone marrow-derived macrophages (BMDM) were isolated from the Trem2-Cko and Trem2-Ctr mice. Briefly the bone marrow was flushed from the tibias and femurs using phosphate-buffered saline (PBS) (G4202, Servicebio, Wuhan, China) containing 100 units/ml penicillin and 100 μg/ml streptomycin (15140-122, Gibco, Grand Island, NY, USA). The bone marrow was resuspended, and the resulting suspension was filtered through a 70-μm cell strainer (352350, BD Falcon, NJ, USA), followed by centrifugation at 1,500 rpm for 5 min. Then, sterilized Red Blood Cell Lysis Buffer (AR1118, Boster Biological Technology, Wuhan, China) was used to remove red blood cells at 4 °C for 5 min followed by centrifuging the medium at 1,500 rpm for 5 min. The cells thus obtained were cultured in Dulbecco’s modified Eagle’s medium (DMEM) (11995-065, Gibco, Grand Island, NY, USA) with 10% (v/v) fetal bovine serum (FBS; 10100147, Gibco, Grand Island, NY, USA), 20 ng/ml macrophage colony-stimulating factor (M-CSF; HZ-1192, Proteintech, Wuhan, China), 100 U/ml penicillin, and 100 μg/ml streptomycin. The cells were allowed to differentiate into macrophages for 7 d at 37 °C in a 5% CO_2_ incubator. To investigate the effects of radiation on macrophages, the 5-Gy x-ray radiation was used as described previously. Macrophages were then incubated for 24 h and harvested for further analysis.

### Cell treatment

RAW264.7 cells were used to detect the effects of TREM2 under irradiation. Briefly, the mouse *Trem2* open reading frame (ORF) cDNA clone, containing a C-GFPSpark tag (MG50149-ACG, Sino Biological, Beijing, China), was transfected into RAW264.7 cells to overexpress TREM2 (OE-Trem2). Cells transfected with the Pcmv3-C-GFPSpark Negative Control Vector (CV026, Sino Biological, Beijing, China) served as the control group. *Trem2* knockdown (si-Trem2) was performed using siRNA, and negative control RNA was generated by Sango Biotech (Shanghai, China). The sequences are as follows: siTrem2-447: (5′-CCAGUGUCAGAGUCUCCGATT-3′; 5′-UCGGAGACUCUGACACUGGTT-3′); siTrem2-543: (5′-GGACCCUCUAGAUGACCAATT-3′; 5′-UGGUCAUCUAGAGGGUCCTT-3′); siTrem2-881: (5′-GGAGGUACGUGAGAGAAUUTT-3′; 5′-AAUUCUCUCACGUACCUCCTT-3′). Transient transfection of RAW264.7 cells with plasmid (2 μg/ml) and siRNA (50 nM) was accomplished using Lipofectamine 3000 Transfection Reagent (L3000075, Invitrogen, CA, USA) according to the manufacturer’s instructions. To inhibit Trem2 cleavage effects, RAW264.7 was preincubated with 200 nM GW280264X (HY-115670, MCE, NJ, USA), an ADAM10/17 inhibitor, in complete medium for 24 h before radiation treatment. Cells and supernatants were collected for analysis.

### Cell viability

Cell viability was estimated using the Cell Counting Kit-8 (C0039, Beyotime, Shanghai, China) according to the manufacturer’s instructions. Cell viability was measured at different time points and normalized to that at the initial time point. A Viability Assay kit (Calcein-AM, EthD-I) (PF00008, Proteintech, Wuhan, China) was used for live/dead cell staining, and IntDen was used to compare cell viability. All groups were observed using an IXplore fluorescence microscope (IX85, Olympus, Japan), and images were analyzed using ImageJ software. As a supplementary observation of growth dynamics, a scratch-based assay was also performed. Briefly, the cells were seeded in the culture plates and sterilized 200-μl tips were used to create uniform scratch at the bottom of the plates. Then, images of the plate were obtained at different time points after subjecting the cells to different treatments. All groups were observed using an IXplore fluorescence microscope (IX85, Olympus, Japan), and images were analyzed using ImageJ software.

### ROS staining

To detect radiation-induced ROS, we used a Reactive Oxygen Species assay kit (BL714A, Biosharp, Hefei, China) according to the manufacturer’s instructions. Briefly, 10 μM 2',7'-dichlorodihydrofluorescein diacetate (H2DCFDA) was preincubated for 30 min before radiation, and cell samples were harvested at 2 and 6 h, after which photos were taken using an IXplore fluorescence microscope. A SpectraMax iD3 multi-mode microplate reader (Molecular Devices, CA, USA) was used to measure the reactions, with fluorescent excitation at 488 nm and emission at 525 nm.

### IF staining

For IF staining, cells were seeded on 14-mm round coverslips and subjected to different treatments. The slips were then fixed with freshly prepared 4% paraformaldehyde for 10 min, followed by permeabilization with 0.2% Triton X-100 (ST795, Beyotime, Shanghai, China) in PBS for 10 min. The slips were then incubated with anti-Nrf2 (1:100, HA721432, Huabio, Hangzhou, China) and anti-TREM2(1:500, ab305103, Abcam, Cambridge, UK) overnight at 4 °C. Following this, CoraLite488-conjugated goat anti-rabbit (1:500, SA00013-2, Proteintech, Wuhan, China) secondary antibody was added followed by a 1-h incubation at 37 °C in the dark. Nuclei were labeled with DAPI Fluoromount-G (0100-20, Southern Biotech, BHM, USA), and the cells were visualized using a fluorescence microscope (IXplore).

### Flow cytometry

The eBioscience Annexin V Apoptosis Detection Kit APC (88-8007-74, Thermo Fisher, MA, USA) was used to detect the cell apoptotic process according to the manufacturer’s instructions. For analyzing macrophage polarization, cells suspensions that had undergone different treatments were stained with eBioscience Fixable Viability Dye (1:200,65-0865-14, Thermo Fisher, MA, USA) and corresponding fluorescent-labeled antibodies—anti-CD86 (1:100,105032, BioLegend, CA, USA), anti-CD206 (1:100, 141706, BioLegend, CA, USA), anti-CD11b (1:200, 101206, BioLegend, CA, USA), and anti-F4/80 (1,100,123116, BioLegend, CA, USA)—were added after dilution in PBS at the indicated concentrations. The results are presented in percentage or the cell number and percentage. All experiments were performed on a Cytek Aurora (Cytek Bioscience, DE, USA) flow cytometer, and data were analyzed using FlowJo software (V10.0.7, USA).

### Quantitative PCR

Total RNA from the cells and tissues was extracted using an EZ-press RNA Purification Kit (B0004DP, EZBioscience, MN, USA) and Universal RNA Purification Kit (EZB-RN4, EZBioscience, MN, USA) following the manufacturer’s instructions. The RNA was reverse-transcribed using an Evo M-MLV RT Premix kit (AG11706, Accurate Biology, Hunan, China), and qPCR was performed using the SYBR Green Premix Pro Taq HS qPCR Kit II (AG11719, Accurate Biology, Hunan, China) on a QuantStudio 5 (Thermo Fisher, MA, USA). β-Actin was used as the internal control for experiments. Each reaction was performed in duplicate, and changes in relative gene expression normalized to internal control levels were determined using the relative threshold cycle method. Primers (forward/reverse): β-actin (5′-GGCTGTATTCCCCTCCATCG-3′/5′-CCAGTTGGTAACAATGCCATGT-3′); Trem2 (5′-CTGGAACCGTCACCATCACTC-3′/5′-CGAAACTCGATGACTCCTCGG-3′); ADAM10 (5′-ATGGTGTTGCCGACAGTGTTA-3′/5′-GTTTGGCACGCTGGTGTTTTT-3′); and ADAM17 (5′-AGGACGTAATTGAGCGATTTTGG-3′/5′-TGTTATCTGCCAGAAACTTCCC-3′).

### Western blot

Protein extraction from cells and tissues was performed using X-RIPA UltraMix (Xblot-012, X-blot Life Science, Suzhou, China), and debris in the lysates was removed via centrifugation. Protein quantification was performed using a BCA kit (G2026, Servicebio, Wuhan, China). Equal amounts of protein from each sample were mixed with 4× Laemmli Sample Buffer (1610747, Bio-Rad, CA, USA) and 100 μM dithiothreitol (GC205010, Servicebio, Wuhan, China) and used for sodium dodecyl sulfate–polyacrylamide gel electrophoresis (PG211, PG212, PG213, Epizyme Biotech, Shanghai, China) to separate proteins. Following this, the separated proteins were transferred to polyvinylidene fluoride membranes (IPVH00010, Merck Millipore, Darmstadt, Germany) and blocked with Protein Free Rapid Blocking Buffer (PS108P, Epizyme Biotech, Shanghai, China). Then, the membranes were incubated with primary antibodies (Table S1) at 4 °C overnight followed by incubation with a secondary antibody (1:10,000, 170841, Jackson, PA, USA) at room temperature for 1 h. All bands were visualized by chemiluminescence (Tanon 6600; Tanon, Shanghai, China).

### Enzyme-linked immunosorbent assay

The expression levels of inflammatory factors IL-1β, TNF-α, IL-6, IL-10, sTREM2, and total CASPASE3 were measured using ELISA kits (JL20435, JL20242, JL20268, JL18442, JL10484, and JL10456, Jonlnbio, Shanghai, China) according to the manufacturer’s instructions. Thoroughly ground tissue samples and cell suspensions were used for this analysis. The optical density was measured at 450 nm (OD_450_) using a SpectraMax iD3 plate reader.

### Bulk RNA sequencing

A mouse model of irradiation was established by subjecting mice to 7 Gy of radiation after skin excision, and the corresponding RNA-seq dataset was derived from the Gene Expression Omnibus (GEO) database (GSE241297). RNA from RAW264.7 and BMDM cells was extracted using TRIzol reagent (15596026, Invitrogen, CA, USA) according to the manufacturer’s instructions. Transcriptome sequencing and analysis were performed by Beijing Tsingke Biotech Co. Ltd. (Beijing, China). Sample quality metrics and raw read counts were imported to R for further processing. The edgeR [83] package was used to perform differential expression analyses, and genes were considered significantly differentially expressed at *P* < 0.05, |Log_2_FoldChange| > 1. Downstream analysis was performed using the GSEABase (version 1.61.0) [84] and GseaVis (version 0.1.0) [85] package. The raw sequence data reported in this paper have been deposited in the Genome Sequence Archive of the National Genomics Data Center [86,87], China National Center for Bioinformation/Beijing Institute of Genomics, Chinese Academy of Sciences (GSA:CRA027386), which is publicly accessible at https://ngdc.cncb.ac.cn/gsa.

### Statistical analyses

All data are presented as mean ± standard deviation (SD) and were tested for normal distribution. Statistical analyses were performed using GraphPad Prism 8.0. The unpaired Student’s *t* test was used to compare 2 groups. For comparisons among more than 2 groups, one-way analysis of variance (ANOVA) followed by Tukey’s post hoc multiple comparisons test was used. Two-way ANOVA was performed to evaluate the effects of radiation and treatment, and to assess potential interaction effects between the 2 factors. When significant differences were detected, Tukey’s post hoc test was applied. A *P* value < 0.05 was considered statistically significant.

## Data Availability

All data supporting the findings of this study are included in the manuscript and the Supplemental Materials.
