# Erratum to “TREM2 Deficiency Regulates Macrophage Apoptosis and Repair in Radiation-Induced Skin Injury”

**DOI:** 10.34133/research.1376

**Published:** 2026-08-05

**Authors:** Zijian Chen, Siyuan Cai, Pengfei Li, Ziyi Zhou, Zhenxing Huang, Juntao Deng, Linbo Jin, Zucheng Luo, Dongli Fan, Junli Zhou, Fazhi Qi, Yiming Zhang

**Affiliations:** ^1^ Department of Plastic and Cosmetic Surgery, Xin Qiao Hospital, Army Medical University, Chongqing 400037, China.; ^2^ Department of Plastic and Reconstructive Surgery, Zhongshan Hospital, Fudan University, Shanghai, China.; ^3^ Department of Gastrointestinal Surgery, Shanghai 4th People’s Hospital, Tongji University, Shanghai 200081, China.; ^4^ Department of Burn and Plastic Surgery, The Tenth Affiliated Hospital, Southern Medical University (Dongguan People’s Hospital), Dongguan, China.

In the originally published version of “TREM2 Deficiency Regulates Macrophage Apoptosis and Repair in Radiation-Induced Skin Injury”, an error was present in Fig. 4, panel E. The statistical graph corresponding to Western blot quantification was inadvertently assembled using the statistical graph from the qPCR analysis. The corrected Fig. 4 is provided below. The correction was made based on the original Western blot images and densitometric quantification data.

In addition, the legend of Figure S9 contained a wording error. The phrase “(B) qPCR analysis of Trem2 relative expression in si-Adam17-771, si-Trem2-661, and si-Trem2-460 groups compared with control (n = 3)” should instead read: “(B) qPCR analysis of Trem2 relative expression in si-Adam17-771, si-Adam17-661, and si-Adam17-460 groups compared with control (n = 3)”.

These corrections do not affect the results, interpretation, or conclusions of the article. Fig. 4 and S9 have now been corrected in the original HTML and PDF versions of the article. [1]

**Fig. 4.**
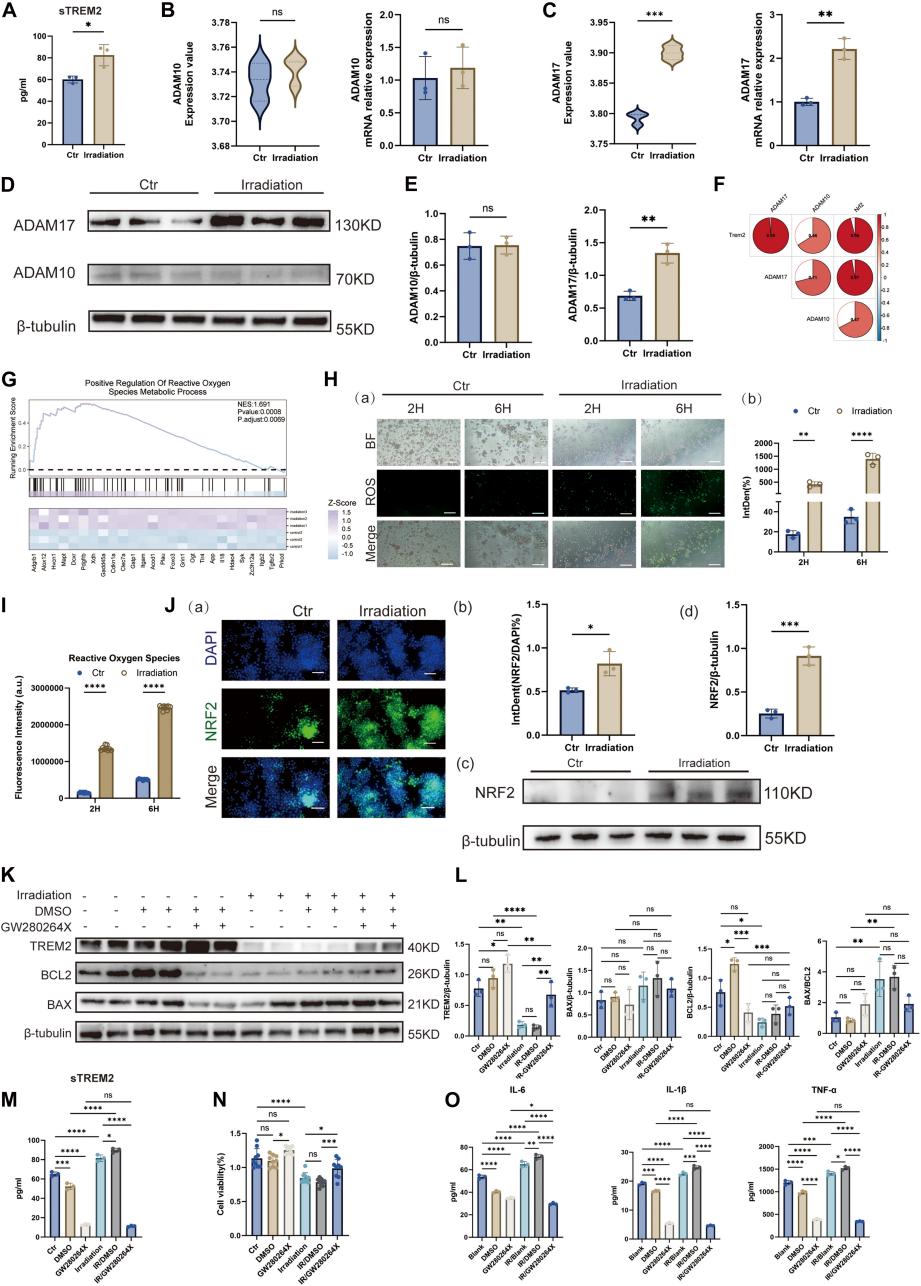
Radiation-induced ROS–Nrf2–ADAM17 axis promotes Trem2 shedding. (A) Quantification of sTREM2 in culture supernatants of RAW264.7 cells under control and irradiated conditions (*n* = 3). (B) ADAM10 transcript levels from RNA-seq and relative mRNA expression measured by qPCR (*n* = 3). (C) ADAM17 transcript levels from RNA-seq and relative mRNA expression measured by qPCR (*n* = 3). (D and E) Western blotting and analysis of ADAM10 and ADAM17 (*n* = 3). (F) Pearson correlation analysis between *Trem2*, *ADAM10*, *ADAM17*, and *Nrf2* based on RNA-seq data. (G) GSEA showing enrichment of the “Positive Regulation of Reactive Oxygen Species Metabolic Process” pathway after irradiation. (H) (a) ROS levels detected by fluorescence staining at 2 and 6 h post-irradiation (green), shown with brightfield (BF) (scale bar, 200 μm); (b) quantification of integrated fluorescence intensity (IntDen) (*n* = 3). (I) ROS quantification based on fluorescence intensity at excitation/emission wavelengths of 488/525 nm (*n* = 3). (J) (a and b) IF detection and quantification of NRF2 (green) with DAPI staining (*n* = 3; scale bar, 200 μm); (c and d) Western blotting and quantification of NRF2 (*n* = 3). (K and L) Western blotting and quantification of TREM2, BAX, and BCL2 protein levels in control, DMSO, and GW280264X cells with or without irradiation (*n* = 3). (M) ELISA detection of sTREM2 in various treatment groups (*n* = 3). (N) Cell viability assay using CCK-8 in control (Ctr), DMSO, and GW280264X (ADAM17/ADAM10 inhibitor) groups with or without irradiation (*n* = 3). (O) ELISA detection of proinflammatory cytokines IL-1β, TNF-α, and IL-6 in various treatment groups (*n* = 3). (*P < 0.05, **P < 0.01, ***P < 0.001, ****P < 0.0001; ns, not significant.).
