# Supplementary material for: TREM2 Deficiency Regulates Macrophage Apoptosis and Repair in Radiation-Induced Skin Injury
Source: Research (Wash D C). 2025 Dec 4;8:1018. doi: 10.34133/research.1018 (PMC12678054; doi:10.34133/research.1018)
Supplement: Supplementary 1 — Materials and Methods Table S1 Figs. S1 to S15 [file research.1018.f1.docx]

**Supplementary materials**

**Trem2 Deficiency Regulates Macrophage Apoptosis and Repair in Radiation-Induced Skin Injury**

*Zijian Chen ^1,2#^, Siyuan Cai ^1#^, Pengfei Li ^2#^, Ziyi Zhou^1^, Zhenxing Huang ^3^, Juntao Deng ^1^, Linbo Jin ^1^, Zucheng Luo ^2^, Dongli Fan ^1^, Junli Zhou^4*^, Fazhi Qi ^2*^, Yiming Zhang ^1*^*

^1^Department of Plastic and Cosmetic Surgery, Xin Qiao Hospital, Army Medical University, Chongqing 400037, China.

^2^Department of Plastic and Reconstructive Surgery, Zhongshan Hospital, Fudan University, Shanghai, China.

^3^Department of Gastrointestinal Surgery, Shanghai 4th People's Hospital, Tongji University, Shanghai, 200081, China

^4^ Department of Burn and Plastic Surgery, The Tenth Affiliated Hospital, Southern Medical University (Dongguan People's Hospital), Dongguan, China.

*Correspond authors:

Junli Zhou: E-mail: [zhoujunli@smu.edu.cn](mailto:zhoujunli@smu.edu.cn);

Fazhi Qi, E-mail: [qi.fazhi@zs-hospital.sh.cn](mailto:qi.fazhi@zs-hospital.sh.cn);

Yiming Zhang, E-mail: zhangyiming@tmmu.edu.cn

# There authors contributed equally to this work.

**This section includes:**

**Table S1**

**Figure S1-S15**

| Primary antibodies | Dilution | Catalog | Company, location |
| --- | --- | --- | --- |
| anti-β-tubulin | 1:2000 | AF1216 | Beyotime, Shanghai, China |
| anti-vinculin | 1:15000 | CY5164 | Abways, Shanghai, China |
| anti-Trem2 | 1:1500 | ab305103 | Abcam, Cambridge, UK |
| anti-BAX | 1:1000 | T40051 | Abmart, Shanghai, China |
| Anti-BCL2 | 1:1000 | T40056 | Abmart, Shanghai, China |
| anti-ADAM10 | 1:1000 | ET1703-60 | Huabio Hangzhou, China |
| anti-ADAM17 | 1:5000 | 84292-4-RR | Proteintech, Wuhan, China |
| anti-Nrf2 | 1:1000 | HA721432 | Huabio, Hangzhou, China |
| anti-caspase9 | 1:1000 | T40046 | Abmart, Shanghai, China |
| anti-pro/cleaved-caspase3 | 1:1000 | ET1608-64 2 | Huabio, Hangzhou, China |
| anti-caspase8 | 1:1000 | ha722482 | Huabio Hangzhou, China |
| anti-Erk1/2 | 1:1000 | T40071 | Abmart, Shanghai, China |
| anti-phospho-Erk1/2 | 1:1000 | T40072 | Abmart, Shanghai, China |
| anti-CD86 | 1:5000 | ET1606-50 | Huabio Hangzhou, China |
| anti-CD206 | 1:1000 | ET1702-04 | Huabio Hangzhou, China |

Table S1 primary antibodies used in western blot


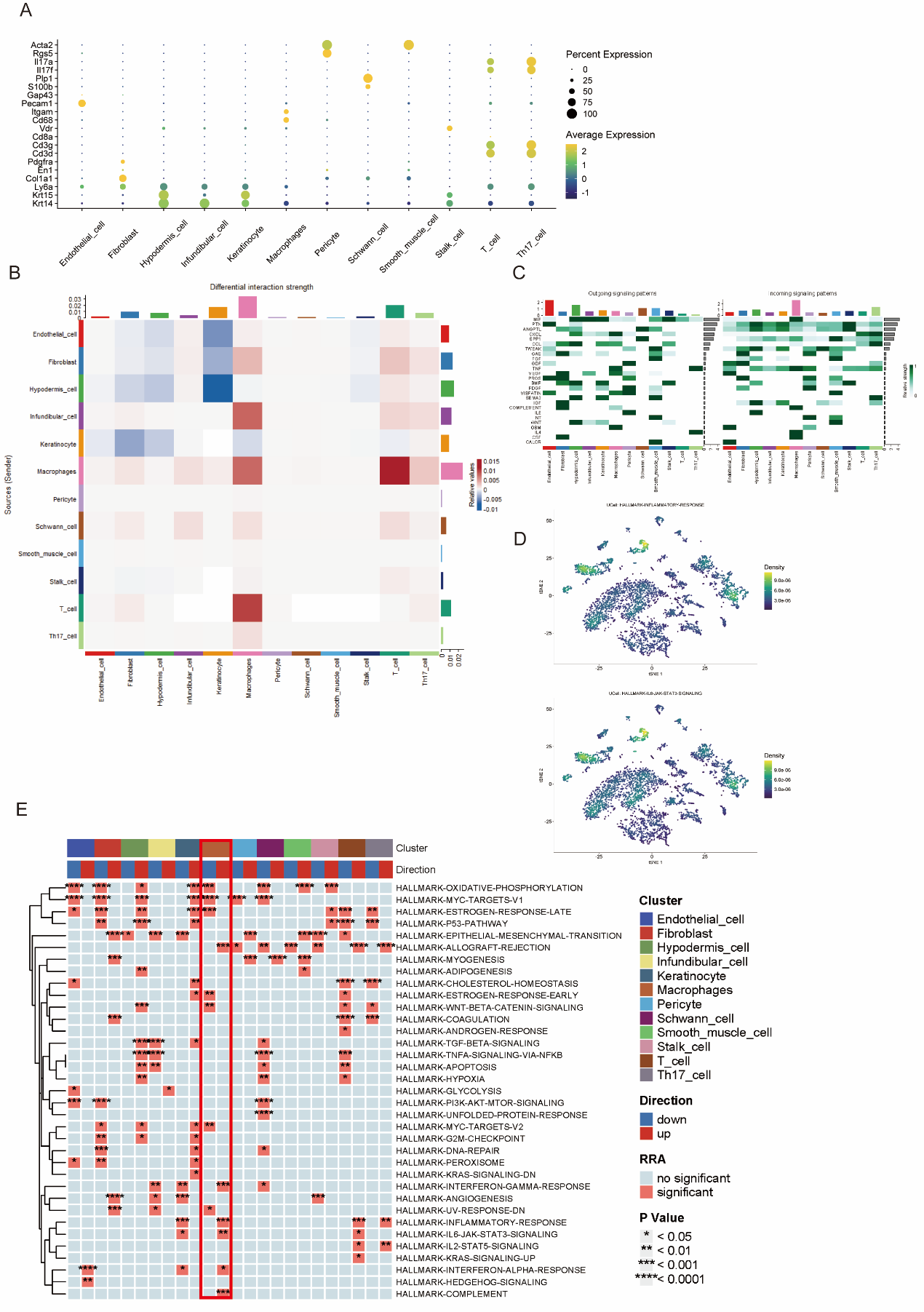


**Figure S1.​​ Single-Cell RNA Sequencing Analysis of radiation skin tissue in mice**
​**​(**A). Cluster annotation based on canonical cell-type markers (t-distributed stochastic neighbor embedding plot). (B). Heatmap of cell differential interaction strength analysis in different clusters. (C). Heatmap of outgoing and incoming signaling patterns in different clusters. (D). UCell enrichment analysis identifying macrophage-dominated clusters (yellow highlight) with significant activity in *inflammatory response* and *IL-6/JAK-STAT3 signaling* pathways. (E). GSEA confirming elevated inflammatory response signature in macrophage clusters (brown).


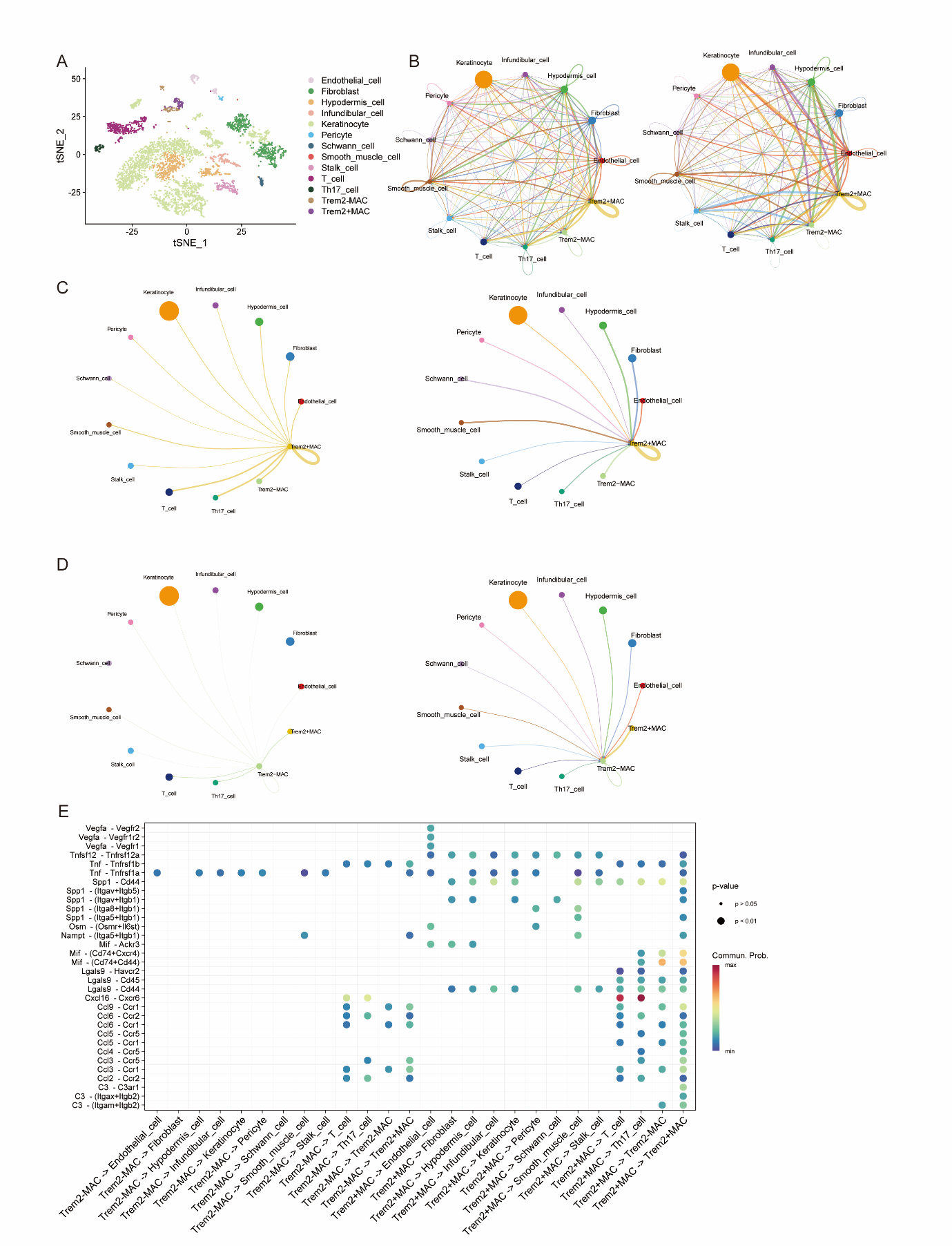


**Figure S2.​​ Single-Cell RNA Sequencing Analysis of radiation skin tissue in mice**
​(A).t-SNE plot showing cluster annotation based on canonical cell-type markers, with integration of TREM2+ and TREM2– macrophage subsets. (B). Chord diagram illustrating the number and strength of cell–cell interactions across different clusters. (C). Chord diagram of interaction numbers and weights for TREM2+ macrophages. (D). Chord diagram of interaction numbers and weights for TREM2– macrophages. (E). Dot plot of intercellular signaling pathways between TREM2+ and TREM2– macrophages.


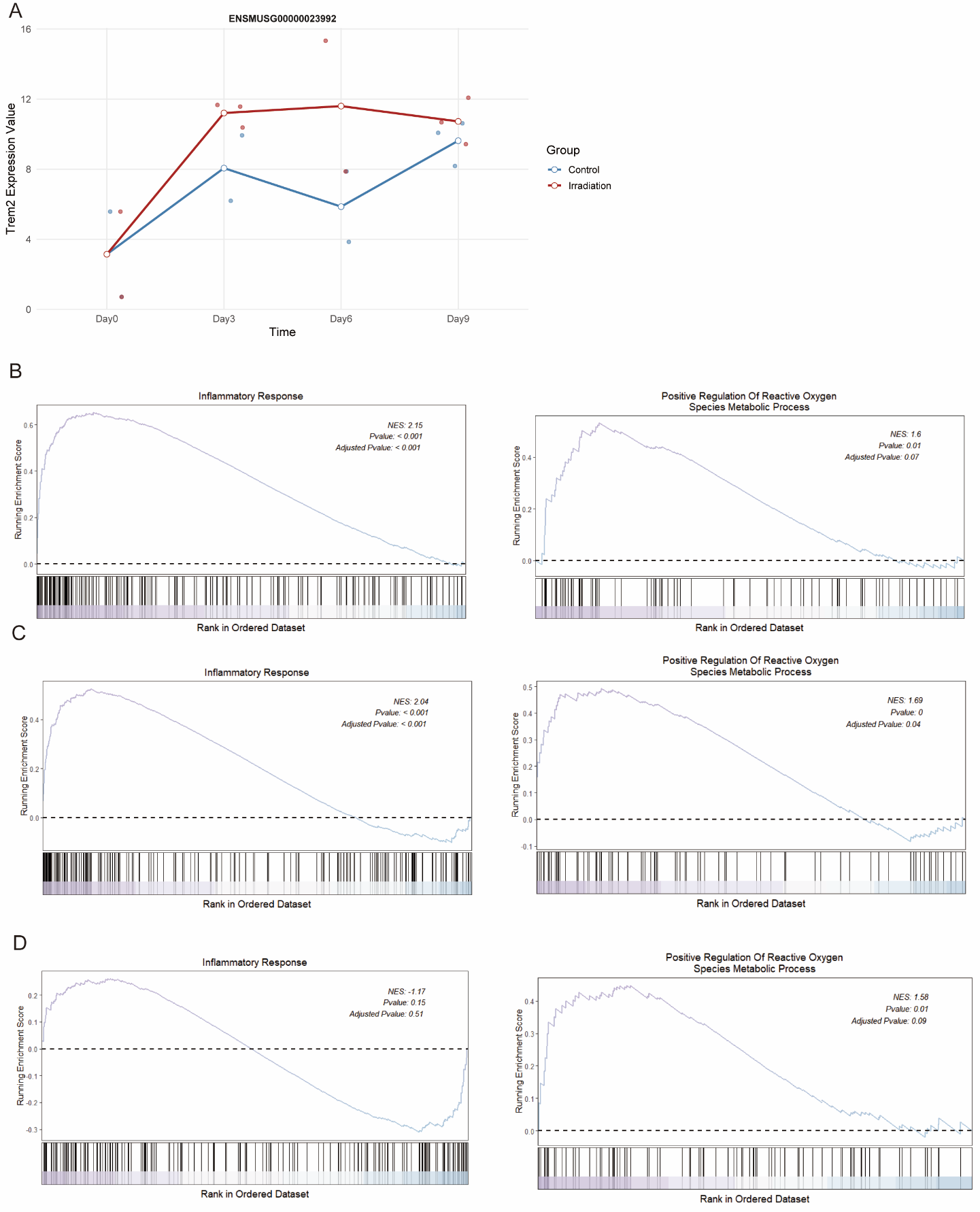


**Figure S3.​​ Bulk RNA Sequencing Analysis of radiation skin tissue comparing with control in mice.**

(A). Temporal expression profile of TREM2 in control and radiation wound groups at Day 0, Day 3, Day 6, and Day 9. (B). Gene set enrichment analysis (GSEA) of differentially expressed genes at Day 3. (C). GSEA at Day 6. (D). GSEA at Day 9.


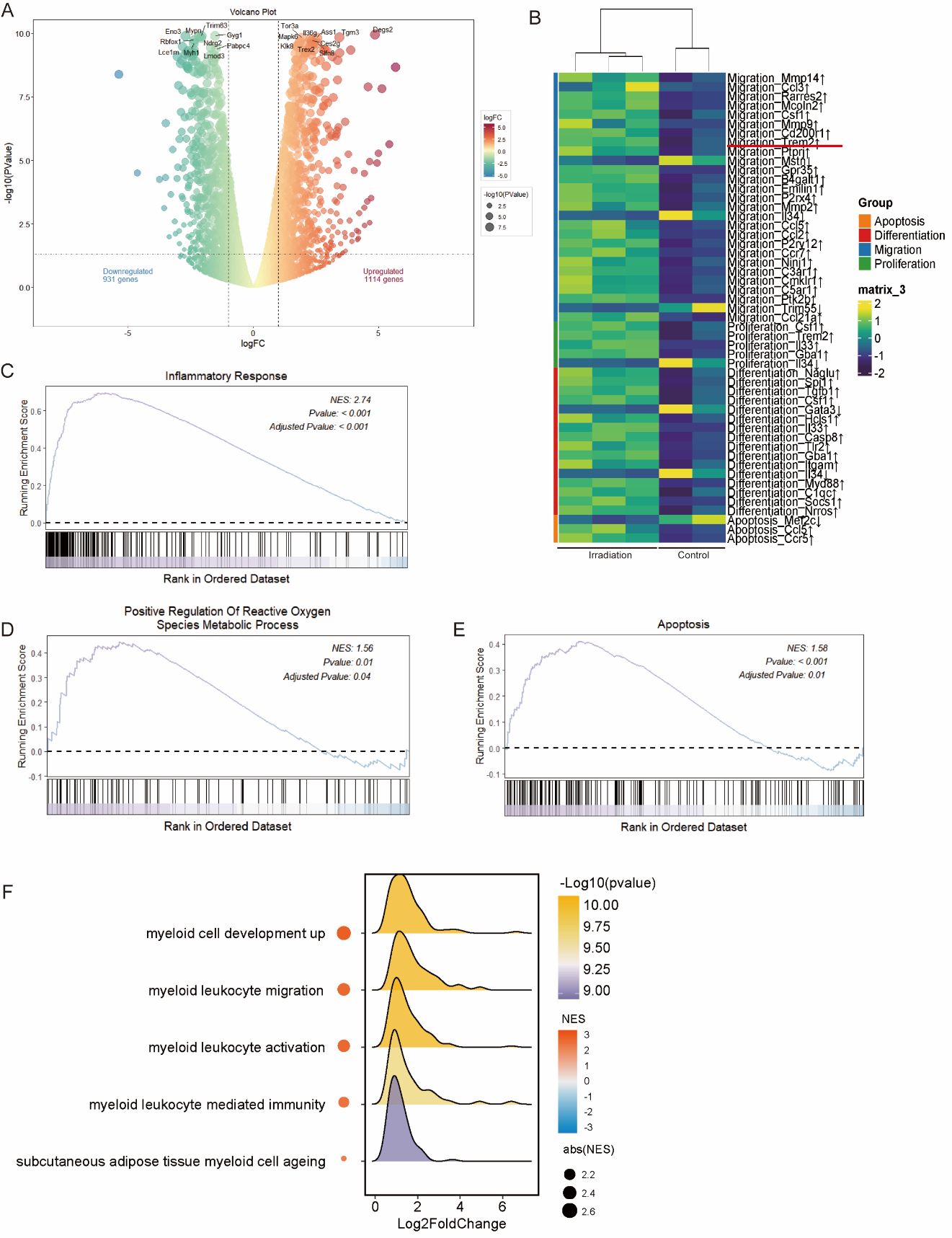


**Figure S4. Bulk RNA sequencing analysis of mouse skin tissue at day 3 post-radiation compared with day 0.**

(A). Volcano plot of differentially expressed genes. (B). Heatmap of apoptosis-, differentiation-, migration-, and proliferation-related genes in myeloid cells. (C). GSEA of inflammatory response. (D). GSEA of positive regulation of reactive oxygen species (ROS) metabolic process. (E). GSEA of apoptosis. (F). GSEA of myeloid cell activity.


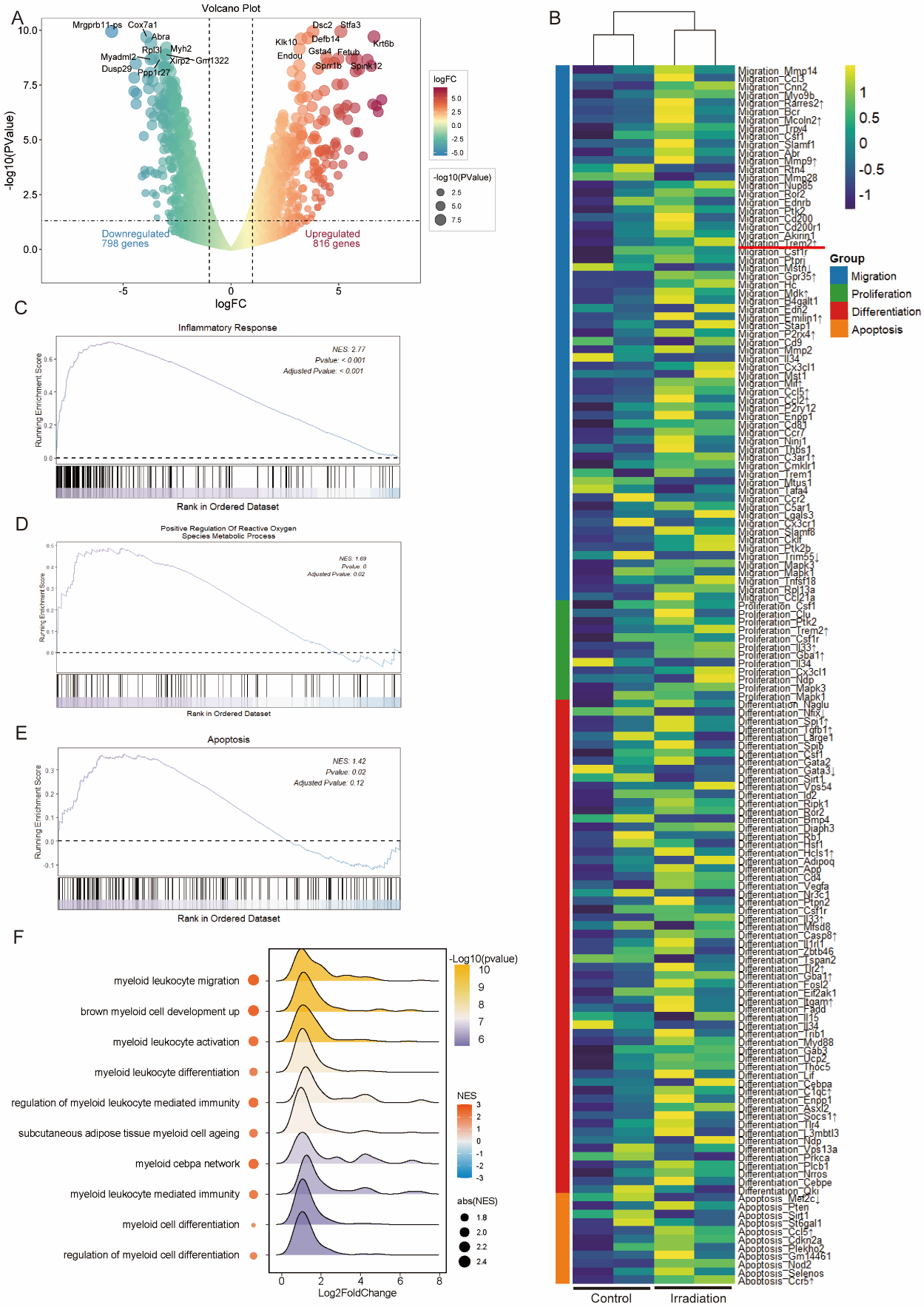


**Figure S5. Bulk RNA sequencing analysis of mouse skin tissue at day 6 post-radiation compared with day 0.**

(A). Volcano plot of differentially expressed genes. (B). Heatmap of apoptosis-, differentiation-, migration-, and proliferation-related genes in myeloid cells. (C). GSEA of inflammatory response. (D). GSEA of positive regulation of reactive oxygen species (ROS) metabolic process. (E). GSEA of apoptosis. F. GSEA of myeloid cell activity.


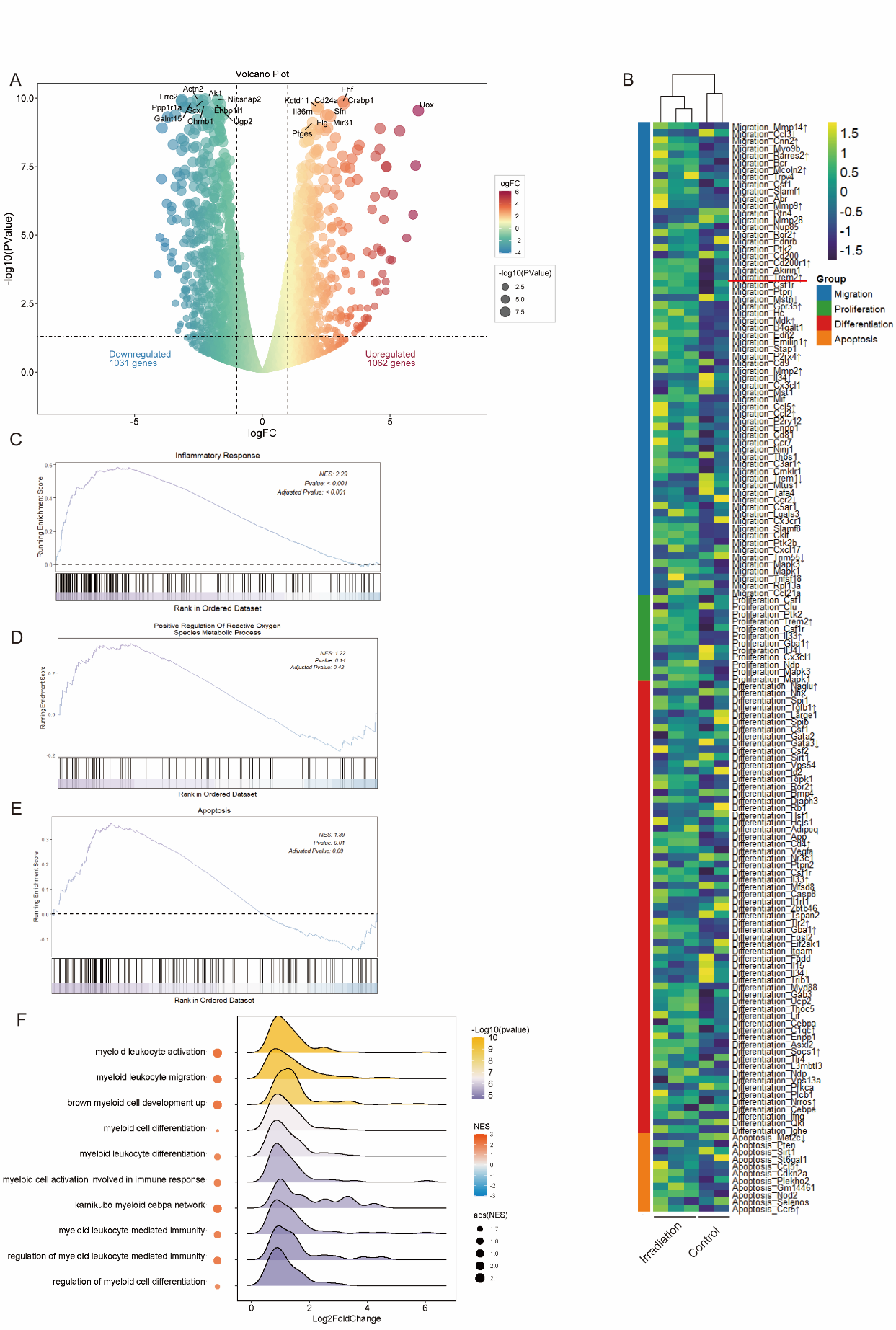


**Figure S6. Bulk RNA sequencing analysis of mouse skin tissue at day 9 post-radiation compared with day 0.**

(A). Volcano plot of differentially expressed genes. (B). Heatmap of apoptosis-, differentiation-, migration-, and proliferation-related genes in myeloid cells. (C). GSEA of inflammatory response. (D). GSEA of positive regulation of reactive oxygen species (ROS) metabolic process. (E). GSEA of apoptosis. (F). GSEA of myeloid cell activity.


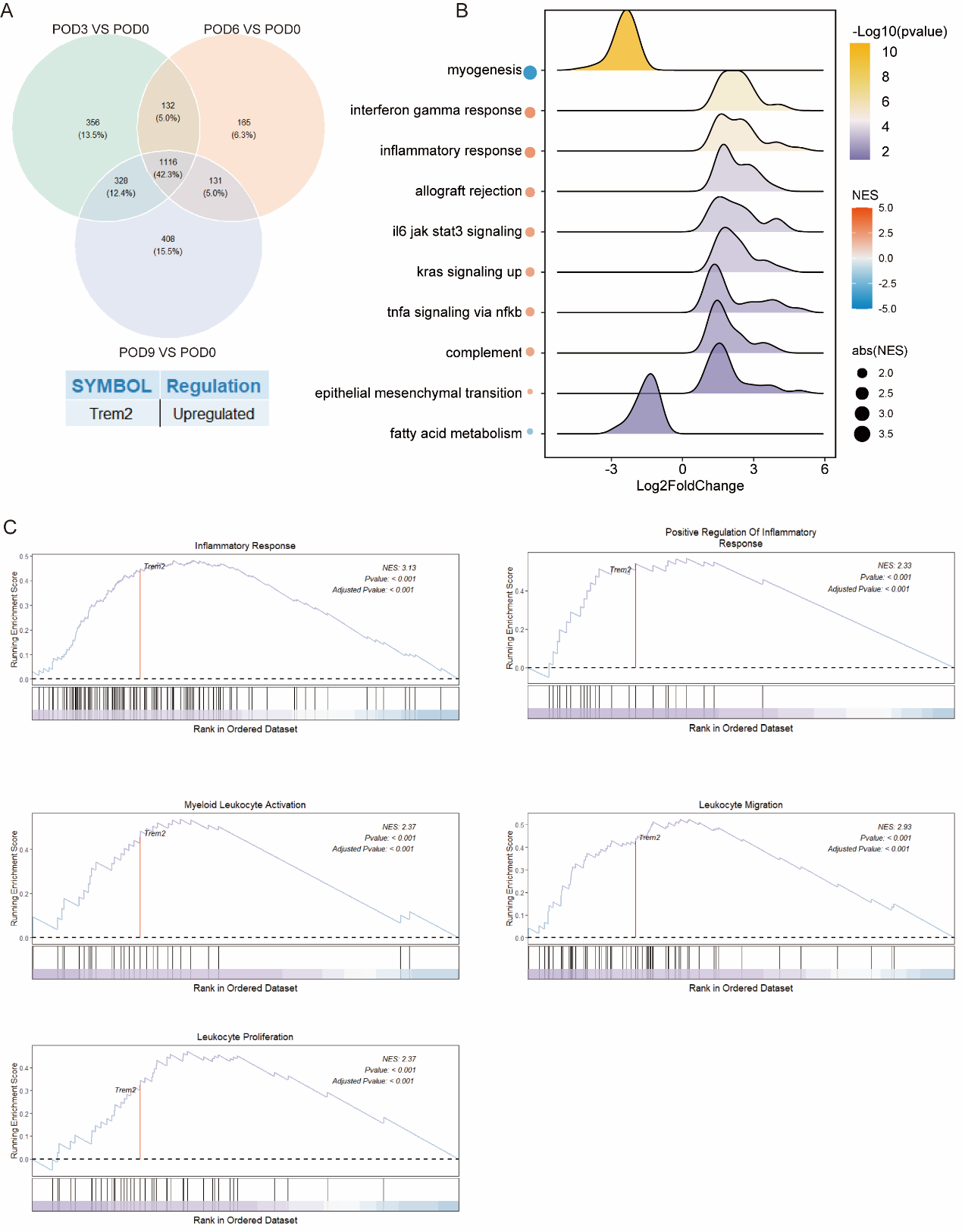


**Figure S7. GSEA of the intersected hub genes derived from comparisons of POD3 vs POD0, POD6 vs POD0, and POD9 vs POD0.**

(A). Venn diagram of hub genes along with the expression profile of TREM2. (B). GSEA of hub genes. (C). GSEA of pathways associated with TREM2 expression.


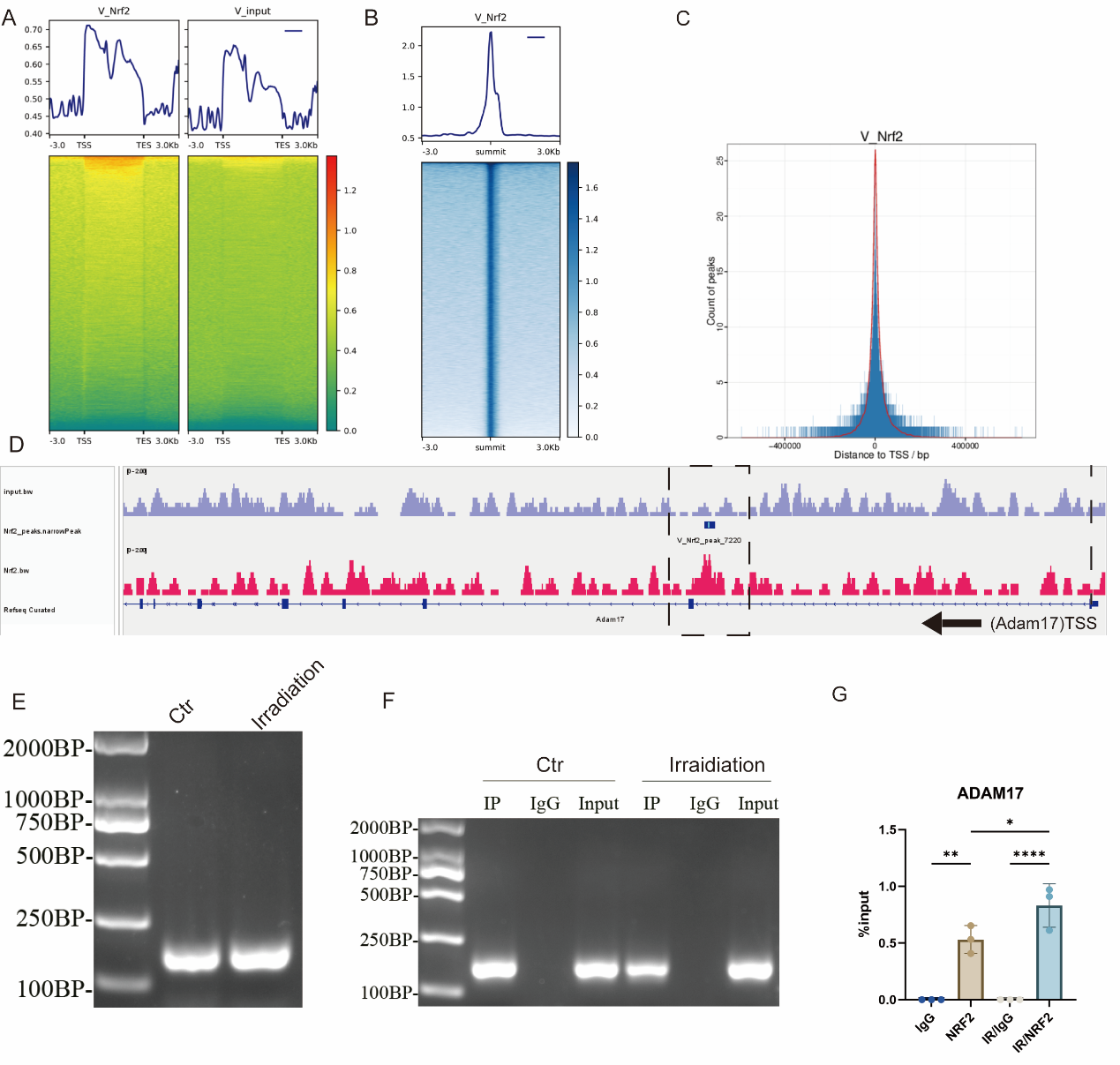


**Figure S8. NRF2 directly promote the ADAM17 transcription as revealed by ChIP-seq and ChIP–qPCR.(A)** Metagene profiles and heatmaps of NRF2 ChIP-seq and input control across transcription start sites (TSS) and transcription end sites (TES), showing global enrichment of NRF2 binding around promoter regions.**(B)** Heatmap centered on NRF2 peak summits.**(C)** Distribution of NRF2 ChIP-seq peaks relative to TSS **(D)** Genome browser (IGV) tracks of NRF2 ChIP-seq at the *ADAM17* locus, highlighting a distinct binding peak around the promoter region (dashed box).**(E)** Agarose gel electrophoresis confirming chromatin fragmentation into ~200–500 bp fragments.**(F)** Conventional PCR detection of the *ADAM17* promoter fragment from ChIP DNA **(G)** ChIP–qPCR quantification of NRF2 binding the *ADAM17* under control(Ctr) and Irradiation conditions, expressed as %Input relative to IgG. (n = 3); (*p < 0.05, **p < 0.01, ****p < 0.0001).


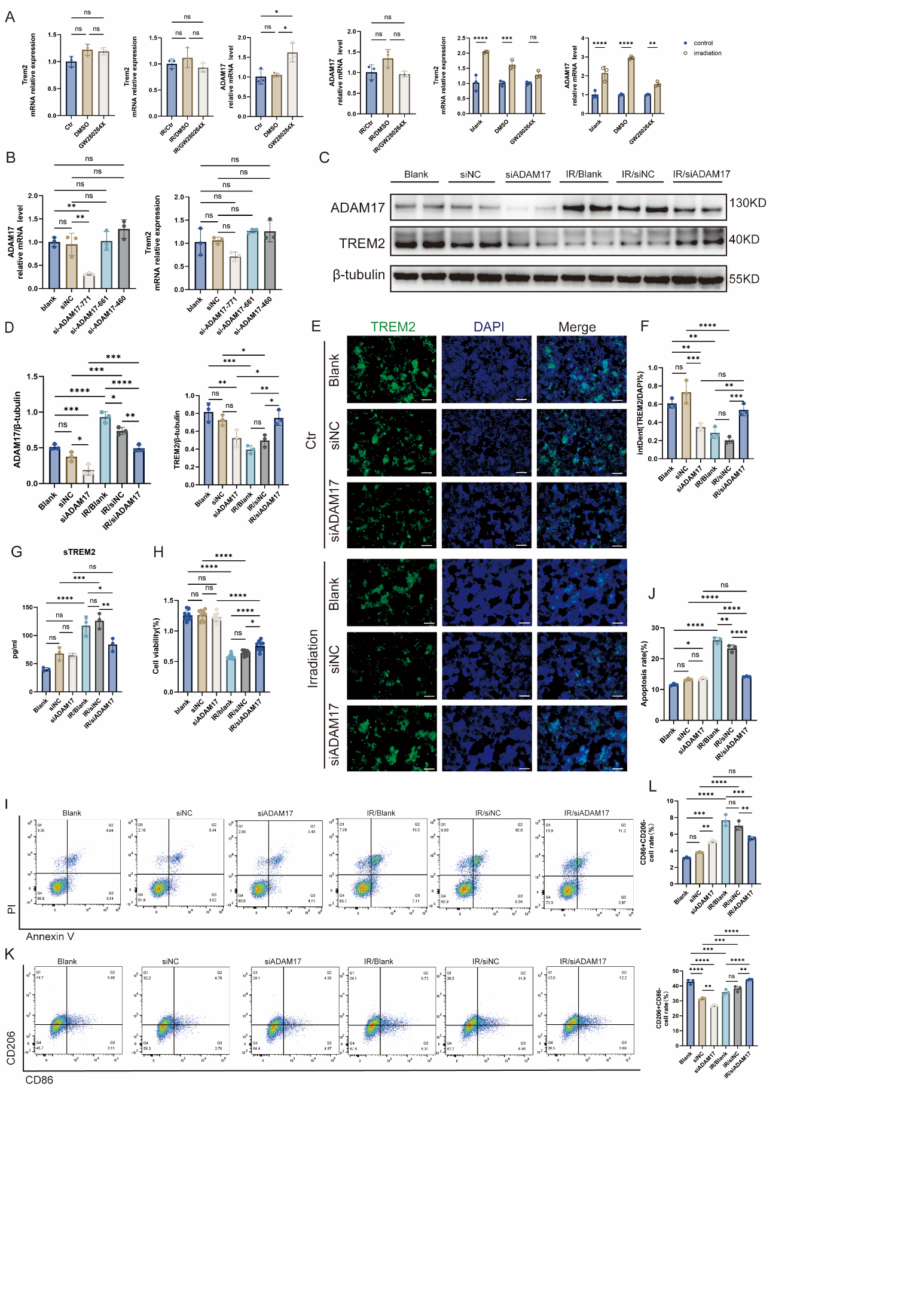


**Figure S9. Effects of ADAM17 knockdown on TREM2 expression and macrophage function.**
(A) Quantitative PCR (qPCR) analysis of ADAM17 and Trem2 expression in GW280264X-treated groups (n = 3). (B) qPCR analysis of Trem2 relative expression in si-Adam17-771, si-Trem2-661, and si-Trem2-460 groups compared with control (n = 3). (C, D) Western blot and quantification of ADAM17 and TREM2 in si-Adam17-771–transfected cells under control and irradiation conditions (n = 3).(E, F) Immunofluorescence detection and quantification of TREM2 (green) with DAPI nuclear staining (n = 3; scale bar = 200 μm).(G) ELISA analysis of sTREM2 in cell culture supernatants (n = 3).(H), Cell viability assay using CCK-8 in Blank, DMSO, and siADAM17groups (n = 3). (I, J) Apoptosis analysis by flow cytometry using Annexin V/PI dual staining (n = 3).(K, L) Flow cytometry analysis of macrophage polarization. M1 polarization was assessed by CD86 expression, and M2 polarization by CD206 expression (n = 3).(*p < 0.05, **p < 0.01, ***p < 0.001, ****p < 0.0001; ns, not significant).


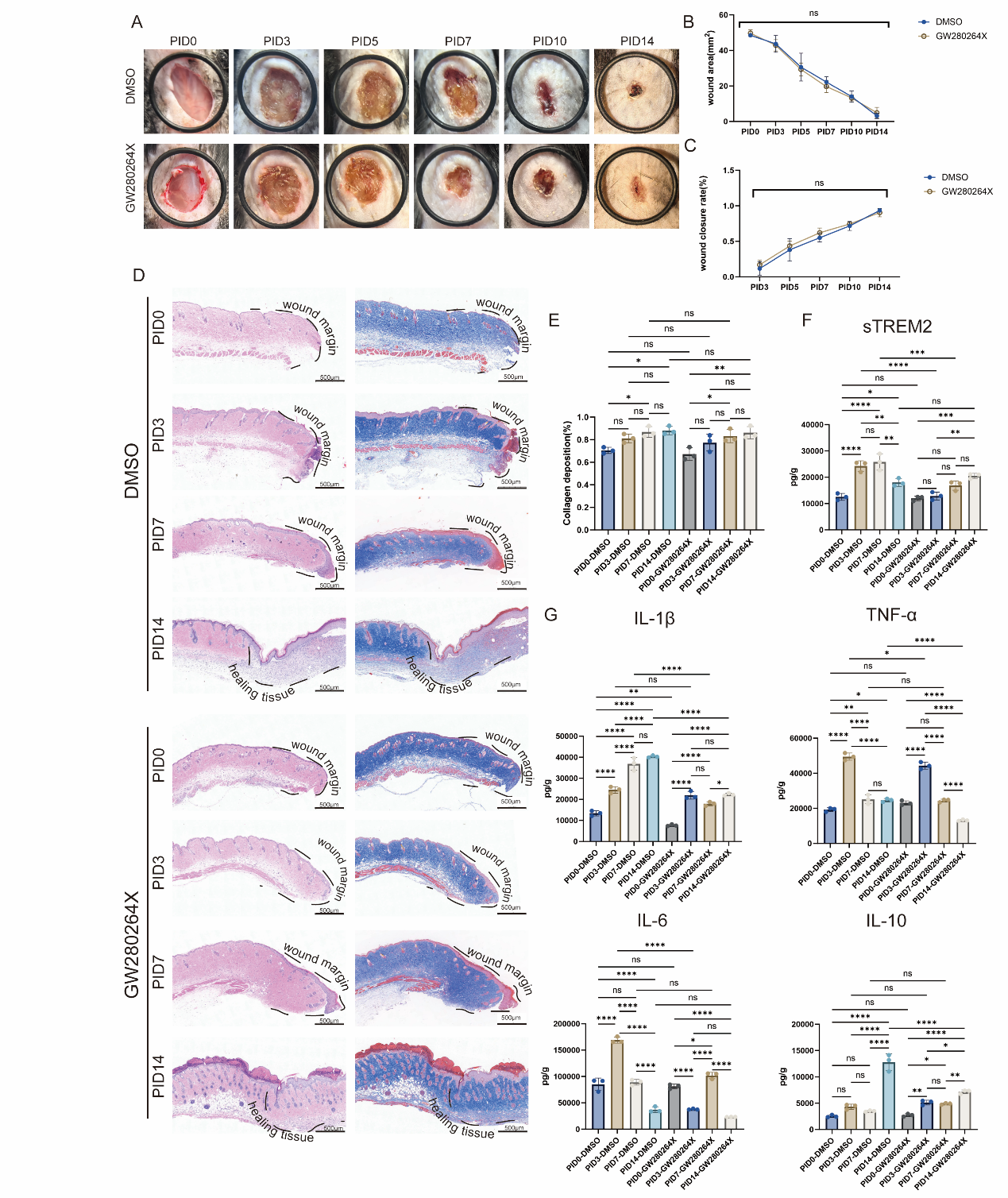


**Figure. S10 Evaluation of the ADAM17 inhibitor (GW280264X) on wound healing under radiation.** (A) Representative wound area measurements (black circle, 12 mm inner diameter = 3).(B,C) Quantification of wound area and closure rates at various time points relative to post-injury day 0 (PID0) (n = 3).(D) H&E and Masson staining of wound tissue sections at PID0, 3, 7, and 14,showing regions close to the wound margin (Scale bar was shown on panel).(E) Quantification of collagen deposition based on Masson staining (n = 3).(F,G) ELISA measurement of soluble TREM2 (sTREM2) , pro-inflammatory cytokines IL-1β, TNF-α, IL-6 and anti-inflammatory cytokines IL-10 levels in skin tissue at different time points in various treatment groups (n = 3) .(*p < 0.05, **p < 0.01, ***p < 0.001, ****p < 0.0001; ns, not significant.)


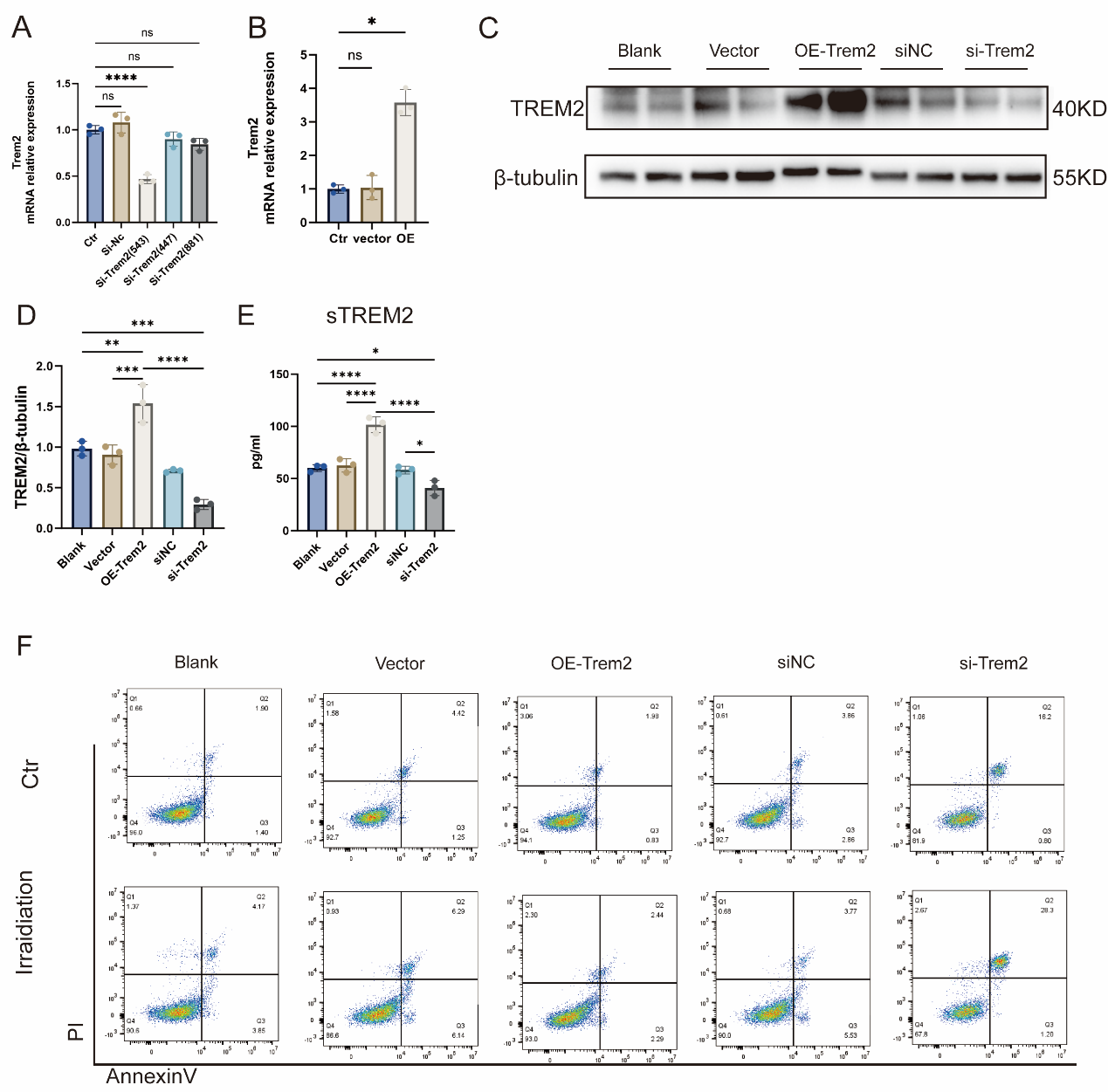


**Figure. S11 Evaluation of Trem2 overexpression and knockdown efficiency and analysis of apoptosis rate​​**
​​(A).​​ Quantitative PCR (qPCR) analysis of Trem2 relative expression in si-Trem2-543, si-Trem2-447, and si-Trem2-881 groups compared to control (Ctr)(n=3).(B).​​ qPCR analysis of *Trem2* overexpression efficiency in the OE-Trem2 group compared to control (Ctr)(n=3).(C, D).​​ Western blot analysis of TREM2 protein expression and quantitative analysis(n=3).​​ (E).ELISA detection of soluble TREM2 (sTrem2) levels(n=3).(F).​​ Apoptosis rate analysis by flow cytometry using Annexin V/PI dual staining(n=3). (*p < 0.05, ** ​​p < 0.01, *** ​​p < 0.001, ****​​​​p < 0.0001; ns, not significant)


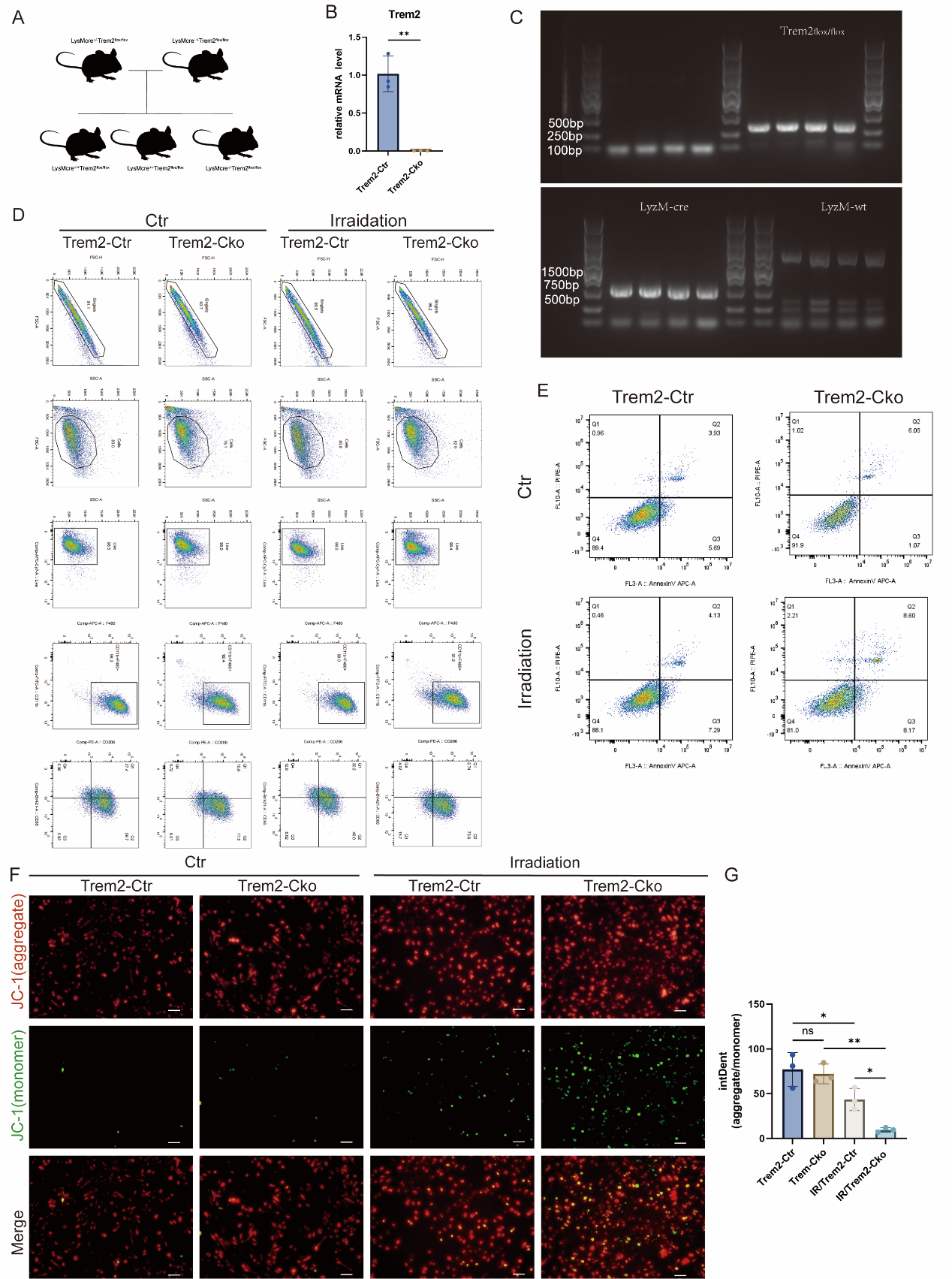


**Figure. S12 Genotyping of LysMcre Trem2^flox/flox^ mice and analysis of macrophage polarization and apoptosis rate​**​
​**​**(A).​​ Schematic representation of Trem2 conditional knockout (Trem2-Cko) and control (Trem2-Ctr) mice. LysM^Cre+^/Trem2^flox/flox^ mice served as Trem2-Cko, and LysM^Cre-^/Trem2^flox/flox^ mice were used as Trem2-Ctr for bone marrow-derived macrophage (BMDM) isolation and *in vivo* studies. (B).​​ qPCR analysis of *Trem2* relative expression in Trem2-Cko BMDMs compared to Trem2-Ctr BMDMs. (C).​​ Genotyping PCR for the Trem2^flox/flox^ (335 bp), LysMCre (543 bp), and wild-type LysM (1413 bp). (D).​​ Flow cytometry analysis of BMDM polarization. Macrophages were gated on CD11b+ F4/80+ populations; M1 polarization was assessed by CD86 expression; M2 polarization was assessed by CD206 (MRC1) expression (n = 3). (E).​​ Apoptosis rate analysis of BMDMs by Annexin V/PI dual staining flow cytometry (n = 3). (F, G) Representative fluorescence images and quantitative analysis of mitochondrial membrane potential assessed by JC-1 staining (n = 3, scale bar = 200 μm.) (*p < 0.05, ** ​​p < 0.01; ns, not significant)


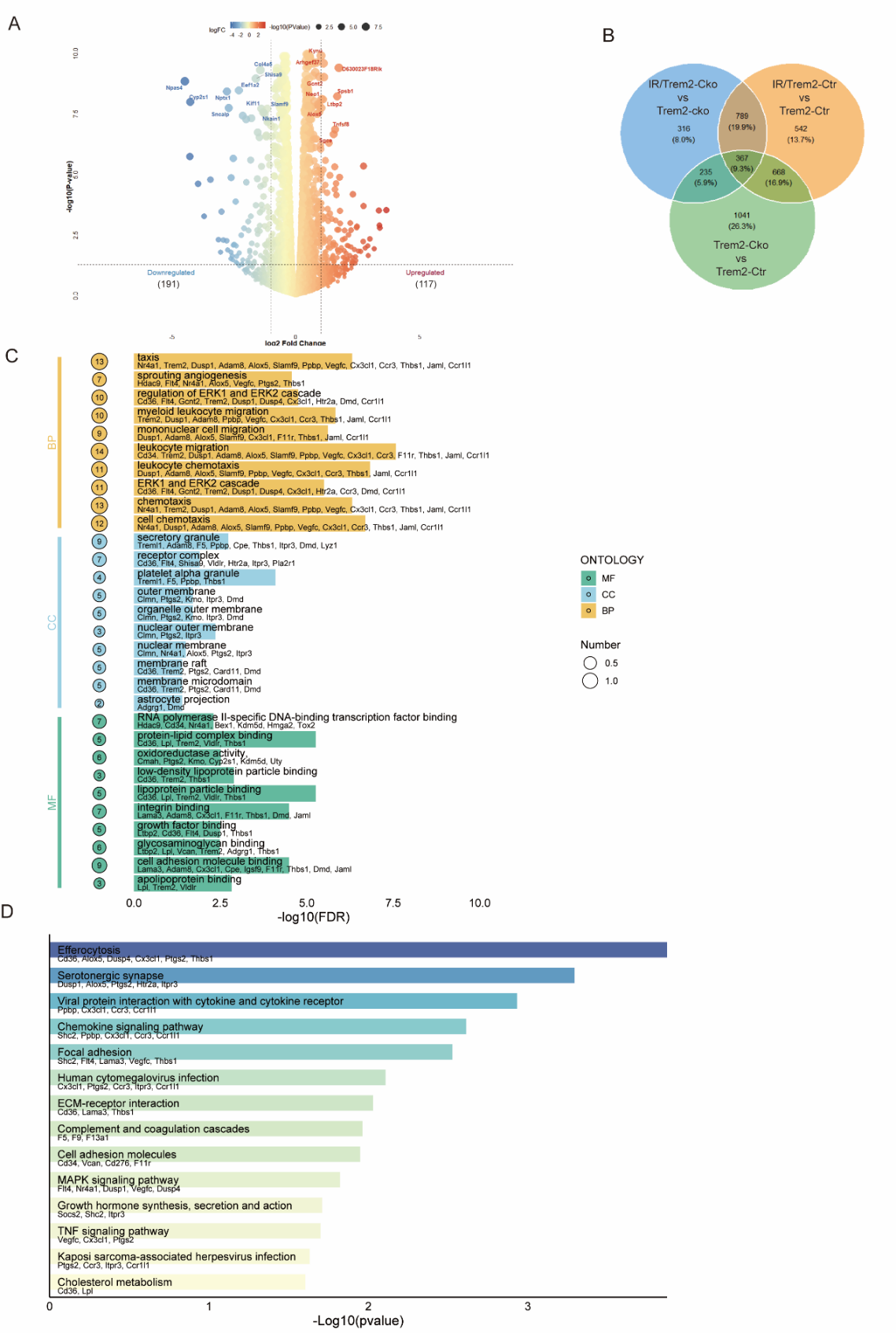
 **Figure. S13 RNA-seq analysis of irradiated Trem2-Cko and Trem2-Ctr BMDMs​**​
​**​A.​**​ Volcano plot of differentially expressed genes (DEGs). 117 upregulated and 197 downregulated genes were identified in IR/Trem2-Cko vs. IR/Trem2-Ctr BMDMs (n = 3). **B.​**​ Integrated analysis of current RNA-seq data and public dataset (CRA01171145) identified 367 hub genes associated with combined Trem2 knockout and radiation effects. Public data included non-irradiated Trem2-Cko and Trem2-Ctr BMDMs.**C.​**​ Gene Ontology (GO) analysis of hub genes showing significant enrichment in "regulation of ERK1 and ERK2 cascade" (Biological Process) following Trem2 deficiency and radiation exposure**.​**​ KEGG pathway analysis demonstrating hub gene enrichment in the MAPK signaling pathway.


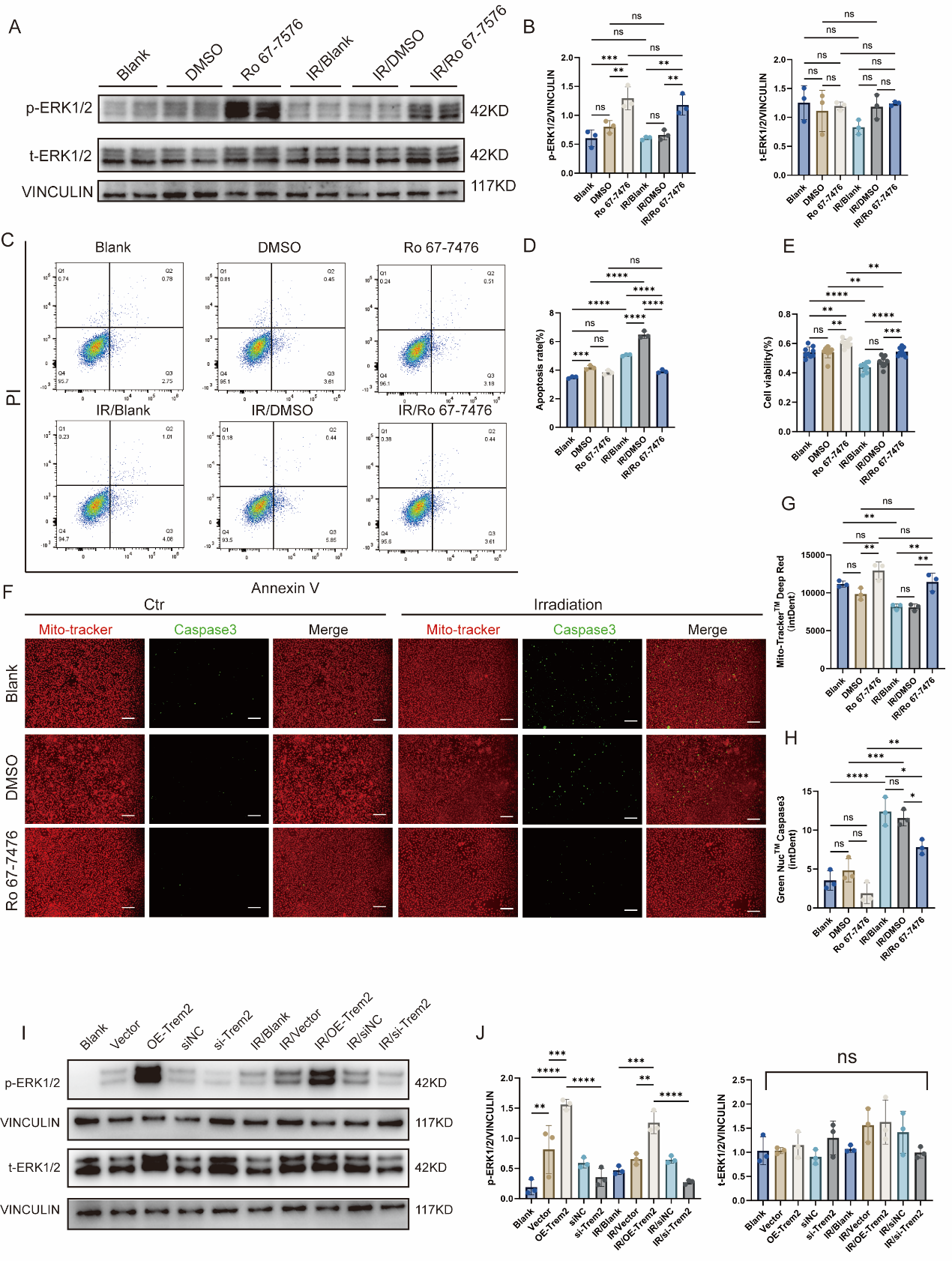


**Figure S14. Rescue of ERK signaling by ERK agonist in Trem2-Cko BMDMs.**(A, B) Western blot and quantification of p-ERK1/2 and total ERK1/2 in Blank, DMSO, and Ro 67-7476 treatment groups (n = 3).(C, D) Apoptosis analysis of BMDMs by Annexin V/PI dual-staining flow cytometry (n = 3).(E) CCK8 assay of cell viability 24 h after irradiation (n = 9).(F–H) Quantification of mitochondrial membrane potential (red fluorescence) and active caspase-3 (green fluorescence) intensity values (n = 3).(I, J) Western blot and quantification of p-ERK1/2 and total ERK1/2 in Trem2-overexpressing or si-Trem2 BMDMs (n = 3).(*p < 0.05, **p < 0.01, ***p < 0.001, ****p < 0.0001; ns, not significant. “ns” not shown in panel J).


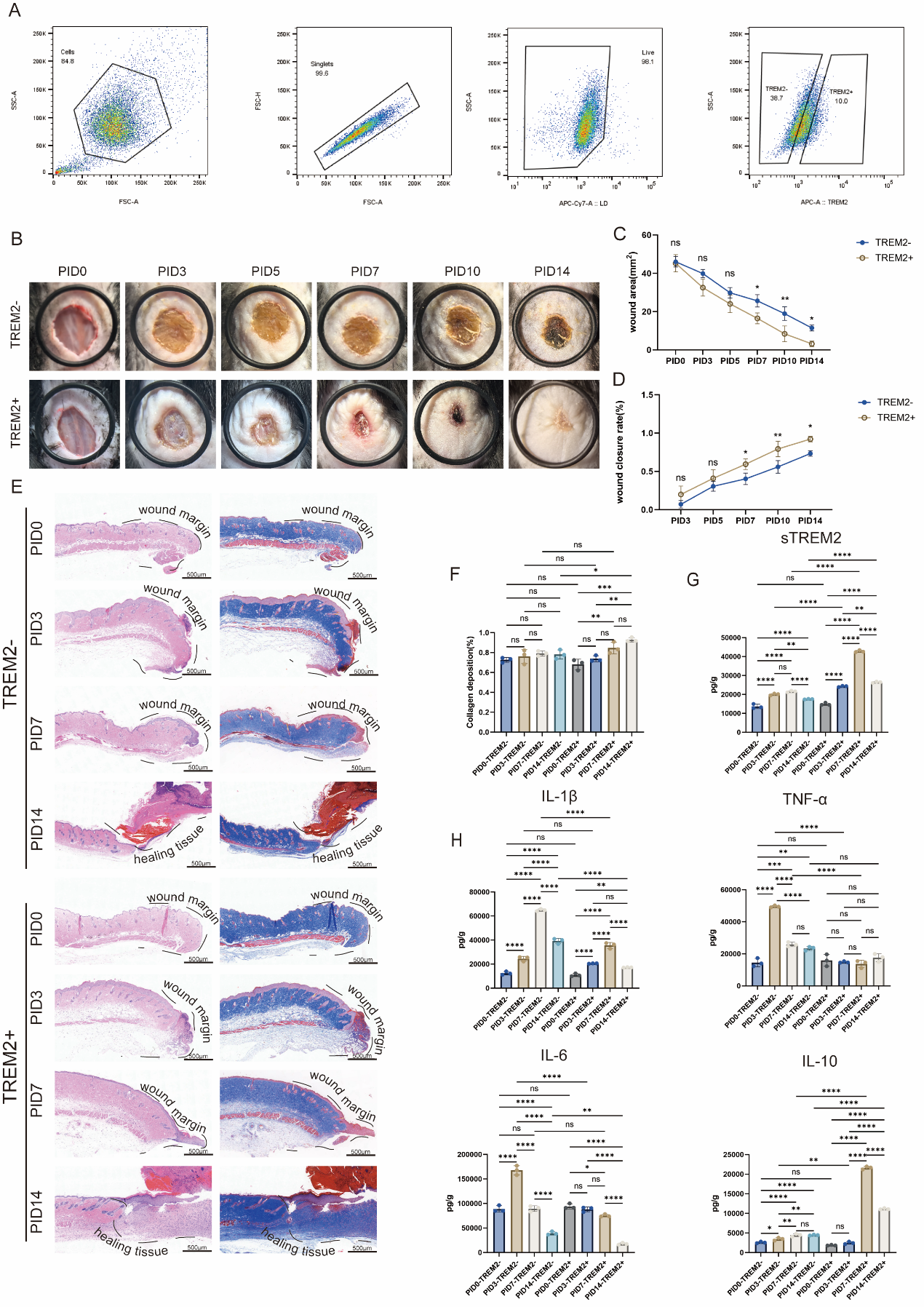


Figure. S15 Impact of TREM2+ and TREM2- macrophage subsets on wound healing under radiation.

(A)Flowcytometry sorting of TREM2+ and TREM2- BMDMs (B) Representative wound area measurements (black circle, 12 mm inner diameter = 3).(B,C) Quantification of wound area and closure rates at various time points relative to post-injury day 0 (PID0) (n = 3).(D) H&E and Masson staining of wound tissue sections at PID0, 3, 7, and 14,showing regions close to the wound margin (Scale bar was shown on panel).(E) Quantification of collagen deposition based on Masson staining (n = 3).(F,G) ELISA measurement of soluble TREM2 (sTREM2) , pro-inflammatory cytokines IL-1β, TNF-α, IL-6 and anti-inflammatory cytokines IL-10 levels in skin tissue at different time points in various treatment groups (n = 3) .(*p < 0.05, **p < 0.01, ***p < 0.001, ****p < 0.0001; ns, not significant.)

**Materials and Methods**

Chromatin immunoprecipitation sequences(ChIP-seq) analysis

ChIP-seq data were obtained from the public GEO dataset GSE221843, which includes NRF2 ChIP-seq samples and corresponding input controls. This dataset was used to analyze NRF2 binding profiles. ChIP-seq libraries were prepared by transposase-mediated fragmentation and adapter ligation, followed by PCR amplification, size selection, and purification. Raw reads were quality-filtered using skewer to remove low-quality and adapter-contaminated sequences. Clean reads were aligned to the reference genome with BWA, and uniquely mapped non-duplicate reads were retained. Peak calling was performed with MACS2 (q ≤ 0.05), and peaks were annotated relative to the nearest transcription start sites using Peak Annotator. Signal enrichment profiles were generated with deep Tools and visualized in ​​Integrative Genomics Viewer (IGV) (version 2.19.6).

Chromatin immunoprecipitation followed by qPCR (ChIP–qPCR)

ChIP assays were performed using RAW264.7 cells (control and irradiated, 5 Gy, 24 h as described before) following standard protocols. Briefly, cells were cross-linked with 1% formaldehyde, lysed, and chromatin was sonicated to ~200–500 bp. Immunoprecipitation was carried out with anti-NRF2 antibody (12721, Cell Signaling Technology, MA, USA) or normal IgG, followed by reverse crosslinking and DNA purification. Input samples were processed in parallel. Purified DNA was analyzed by qPCR using SYBR Green Master Mix (G3322, Servicebio, Wuhan, China). Primers were designed below:

ADAM17(5′-CTGGCATCTAATTTGTGGGG-3′;5′-GTTCGTTGTGGAATGTCTTG-3′), with distal genomic regions as negative controls. Data were normalized to input DNA and expressed as fold enrichment relative to IgG.

Cell treatment

*Adam17* knockdown (si*-Adam17*) was achieved using small interfering RNAs (siRNAs) synthesized by Sango Biotech (Shanghai, China). The sequences were as follows:

si-Adam17-460 :(Sense: 5’-GUUUGUCAAUGAUACUAAA-dTdT-3’; antisense: 5’-UUUAGUAUCAUUGACAAAC-dTdT-3’), si-Adam17-661:(Sense: 5’-GAAGAAUACUUGUAAAUUA-dTdT-3’; antisense: 5’-UAAUUUACAAGUAUUCUUC-dTdT-3’), si-Adam17-771 (sense: 5’-GGGACAGAGUGACGACCGA-dTdT-3’; antisense: 5’-UCGGUCGUCACUCUGUCCC-dTdT-3’). Transient transfection of RAW264.7 cells with siRNAs (50 nM) was performed using Lipofectamine™ 3000 Transfection Reagent (L3000075, Invitrogen, CA, USA) according to the manufacturer’s protocol. To assess ERK1/2 activation, Trem2-Cko BMDMs were treated with Ro-67-7476 (HY-100403, MCE, NJ, USA), a potent ERK1/2 agonist that promotes ERK1/2 phosphorylation. Cells were incubated with 100 nM Ro-67-7476 in complete medium for 24 h following irradiation. Cells and supernatants were then harvested for subsequent analyses.

ADAM17 inhibitor treatment in vivo

Eighteen C57BL/6 mice were randomly assigned into two groups. All mice received 5 Gy whole-body irradiation as previously described, followed by the creation of an 8 mm full-thickness excisional wound on the dorsal skin. The ADAM17 inhibitor GW280264X was first dissolved in DMSO and then diluted with 0.9% sodium chloride to a final concentration of 10 nM. Mice in the treatment group were intraperitoneally injected with GW280264X (vehicle concentration containing 0.0025% DMSO), while the control group received equal volumes of 0.9% sodium chloride with 0.0025% DMSO. Injections were performed daily from day 0 to day 5. Wound healing was assessed over a 14-day period. Photographs were taken on days 0, 3, 7, 10, and 14 to calculate wound closure rates. In addition, wound-adjacent tissues were collected on days 0, 3, 7, and 14 for molecular and histological analyses.

Fluorescence-Activated Cell Sorting (FACS)​​

​​BMDMs were isolated from wild-type C57BL/6 mice as previously described and resuspended in FACS buffer (PBS supplemented with 2% FBS). The cells were then stained with Fixable Viability Dye eF780(1:200,65-0865-14, Thermo Fisher, MA, USA) Human/Mouse TREM2 APC-conjugated Antibody (1:100, FAB17291A, R&D Systems, MN, USA) for 30 minutes on ice in the dark. After staining, the cells were washed twice with FACS buffer. Fluorescence-activated cell sorting was performed on a BD FACSAria III (BD Biosciences, CA, USA). The Trem2-positive population was sorted relative to the unstained control. Sorted cells were collected directly into complete growth medium for subsequent experiments. ​​Data were analyzed using FlowJo software (V10.0.7, USA).

Trem2⁺ macrophage transplantation in vivo

BMDMs were isolated from wild-type C57BL/6 mice as previously described. Trem2⁺ and Trem2⁻ subsets were sorted by described before. Eighteen C57BL/6 mice were randomly divided into two groups. After exposure to 5 Gy whole-body irradiation, mice underwent an 8 mm full-thickness dorsal excisional wound. For cell transplantation, macrophages were resuspended in sterile PBS at a concentration of 5 × 10^6 cells/mL. Each mouse received a total of 2 × 10^5 cells (40 μL suspension) via subcutaneous injection, distributed equally across four sites surrounding the wound using an insulin syringe. The experimental group received Trem2⁺ macrophages, while the control group was injected with Trem2⁻ macrophages at the same dose. Cell transplantation was performed immediately following wound establishment. Wound healing was monitored for 14 days, with photographs obtained on days 0, 3, 7, 10, and 14 for wound closure analysis. Wound-adjacent tissues were collected on days 0, 3, 7, and 14 for subsequent histological and molecular studies.

Genotyping of LysMcre Trem2^flox/flox^ mice

Genomic DNA was isolated using the Triumfi Mouse Tissue Direct PCR Kit (SD312, Genesand Biotech, Beijing, China) according to the manufacturer's protocol. *Briefly*, 2-mm tail tips were incubated in lysis buffer at 55°C for 30 min followed by 98°C for 3 min. After centrifugation at 12,000 rpm for 5 min, the supernatant served as template for PCR amplification.

The following primers (10 μM each) were used:

Trem2 flox:

Forward: 5’-CCATTTTGGCTTGTAGCGTCG-3’

Reverse: 5’-GCTTGCCCGTGAGGGATTTT-3’

LysMCre:

Forward:5’-AGTGCTGAAGTCCATAGATCGG-3’

Reverse:5’-CTGATTCTCCTCATCACCAGG-3’

Wild-type LysM (LysMwt):

Forward: 5’-AGTGCTGAAGTCCATAGATCGG-3’

Reverse: 5’-GTCACTCACTGCTCCCCTGT-3’

Gapdh (control):

Forward: 5’-AGCTTCGGCACATATTTCATCTG-3’

Reverse: 5’-CGTTCACTCCCATGACAAACA-3’

PCR products were resolved on 1% agarose gels (111860, BIOWEST, Spain) prepared with TAE buffer (BL533A, BioSharp, Hefei, China) and stained with DNA Stain (1:10,000, BR1002553, Bioleaper, Shanghai, China). DNA bands were visualized under UV light using the Tanon 6600 chemiluminescence imaging system (Tanon, Shanghai, China)

Mitochondrial function assay

Mitochondrial membrane potential was assessed using a JC-1 assay kit (E-CK-A301, Elabscience Biotechnology, Wuhan, China) according to the manufacturer’s instructions. Briefly, Trem2-Ctr and Trem2-Cko BMDMs were prepared as previously described and seeded into 24-well culture plates. Twenty-four hours after exposure to 5 Gy irradiation, cells were incubated with JC-1 working buffer for 20 min at 37 °C. Fluorescence images were acquired with an IXplore fluorescence microscope (IX85, Olympus, Japan) and analyzed using ImageJ software. Mitochondrial function was evaluated by calculating the IntDent ratio of red (aggregated JC-1) to green (monomeric JC-1) fluorescence.

To further examine the role of ERK activation under Trem2 deficiency, a live-cell mitochondrial membrane potential and caspase-3 activity assay kit (C1073M, Beyotime, Shanghai, China) was employed following the manufacturer’s protocol. Trem2-Cko BMDMs were seeded in 24-well culture plates and, 24 h after irradiation (5 Gy), cells were incubated with staining working buffer for 30 min at 37 °C. Fluorescence was detected using the IXplore fluorescence microscope, and ImageJ was used for quantification. Mitochondrial membrane potential was evaluated based on red fluorescence IntDent, while active caspase-3 activity was assessed based on green fluorescence IntDent.
